# In Silico Identification and Clinical Validation of a Novel Long Non-Coding RNA/mRNA/miRNA Molecular Network for Potential Biomarkers for Discriminating SARS CoV-2 Infection Severity

**DOI:** 10.3390/cells10113098

**Published:** 2021-11-09

**Authors:** Sara H. A. Agwa, Hesham Elghazaly, Mahmoud Shawky El Meteini, Sherif M. Shawky, Marwa Ali, Aya M. Abd Elsamee, Safa Matbouly Sayed, Nadine Sherif, Howida M. Sharaf, Mohamed A. Alhadidy, Marwa Matboli

**Affiliations:** 1 Pathology and Molecular Genomics Unit of Medical Ain Shams Research Institute (MASRI), Faculty of Medicine, Ain Shams University, Cairo 11591, Egypt; 2 Oncology Department, Medical Ain Shams Research Institute (MASRI), Faculty of Medicine, Ain Shams University, Cairo 11382, Egypt; heshamelghazaly@med.asu.edu.eg; 3 Department of General Surgery, School of Medicine, University of Ain Shams, Cairo 11591, Egypt; mahmoud_elmeteini@med.asu.edu.eg; 4 Biochemistry Department, Faculty of Pharmacy, Misr University for Science and Technology, Al-Motamayez District, P.O. BOX 77, 6th October City 12566, Egypt; 5 Center of Genomics, Helmy Medical Institute, Zewail City of Science and Technology, Ahmed Zewail Road, October Gardens, 6th of October City 12578, Egypt; 6 Medicinal Biochemistry and Molecular Biology Department, Faculty of Medicine, Ain Shams University, Cairo 11591, Egypt; marwaali198@yahoo.com (M.A.); emp17.nadine.s.elzahwy@gmail.com (N.S.); 7 Biochemistry and Molecular Genomics Unit of Medical Ain Shams Research Institute (MASRI), Ain Shams University, Cairo 11591, Egypt; aya_ana2025@yahoo.com; 8 Pediatric Department, School of Medicine, Ain Shams University Hospitals, Cairo 11566, Egypt; safamatboly@yahoo.com; 9 Clinical & Molecular Pathology, Ain Shams University Hospitals, Cairo 11591, Egypt; howidasharaf@yahoo.com; 10 Department of Anesthesiology, Intensive Care & Pain Management, Faculty of Medicine, Ain Shams University, Abbassia, Cairo 11591, Egypt; Mohamed_alaaeldin@med.asu.edu.eg

**Keywords:** circulating RNAs, bioinformatics, SARS-COV-2 infection severity prediction, interleukins genetic network, coding and non-coding RNAs, COVID-19 infection potential biomarker, IL11RA protein overexpression

## Abstract

(1) Background: The coronavirus (COVID-19) pandemic is still a major global health problem, despite the development of several vaccines and diagnostic assays. Moreover, the broad symptoms, from none to severe pneumonia, and the various responses to vaccines and the assays, make infection control challenging. Therefore, there is an urgent need to develop non-invasive biomarkers to quickly determine the infection severity. Circulating RNAs have been proven to be potential biomarkers for a variety of diseases, including infectious ones. This study aimed to develop a genetic network related to cytokines, with clinical validation for early infection severity prediction. (2) Methods: Extensive analyses of in silico data have established a novel IL11RA molecular network (IL11RNA mRNA, LncRNAs RP11-773H22.4 and hsa-miR-4257). We used different databases to confirm its validity. The differential expression within the retrieved network was clinically validated using quantitative RT-PCR, along with routine assessment diagnostic markers (CRP, LDH, D-dimmer, procalcitonin, Ferritin), in100 infected subjects (mild and severe cases) and 100 healthy volunteers. (3) Results: IL11RNA mRNA and LncRNA RP11-773H22.4, and the IL11RA protein, were significantly upregulated, and there was concomitant downregulation of hsa-miR-4257, in infected patients, compared to the healthy controls, in concordance with the infection severity. (4) Conclusion: The in-silico data and clinical validation led to the identification of a potential RNA/protein signature network for novel predictive biomarkers, which is in agreement with ferritin and procalcitonin for determination of COVID-19 severity.

## 1. Introduction

Human coronaviruses are newly recognized airborne viruses (family coronaviridae, genus Betacoronavirus) of zoonotic origin. They are positive-stranded RNA viruses with nucleocapsids, and are considered some of the largest RNA viruses, having about 26 to 32 kilobases of RNA. They mainly cause severe acute respiratory syndrome (SARS) [1]. The first coronavirus outbreak appeared in China in 2003, along with four other countries [2]. This newly identified coronavirus was called SARS-CoV-1. Moreover, a second outbreak caused by another species of coronavirus was discovered in Saudi Arabia in 2011, called Middle East respiratory syndrome (MERS-CoV) [3]. At the end of 2019 in Wuhan, Hubei, China, a novel third coronavirus outbreak was reported, caused by severe acute respiratory syndrome coronavirus 2 (SARS-CoV-2). The disease is known as coronavirus disease 2019 (COVID-19) [2,4]. On 11th March 2020, the World Health Organization (WHO) announced COVID-19 as a global pandemic [5]. Before the 6 June 2021, there were over 172.5 million cases reported, and over 3.5 million deaths globally [6].

The clinical picture of COVID-19 cases varies markedly among patients: from asymptomatic to severe pneumonia and death [6]. Clinical features/laboratory investigation-based classifications have been proposed by Yuki et al. [7].

The blood levels of interleukin 2 receptor (IL-2R), IL-6, IL-8 and IL-10 were significantly more upregulated in deceased patients than in recovered ones [8]. Notably, the levels of these serum cytokines were higher in severe SARS-CoV-2-positive cases compared with moderate cases, highlighting the necessity of IL-6 detection for the early prediction of the infection severity [9]. IL-6 and IL-11 both signal through a homodimer of the ubiquitously expressed β-receptor glycoprotein 130 (gp130) [10]. An individual IL-6/IL-11 α-receptor causes the initial binding of cytokine to the α-receptor, leading to the final formation of a complex with the β-receptors. Both cytokines activate the same downstream signaling pathways, primarily the mitogen-activated protein kinase (MAPK) cascade and the Janus kinase/signal transducer and activator of transcription (Jak/STAT) pathway [11]. Several studies refer to IL-6 as having a much broader expression profile of IL-11R compared to IL-6R [10,12]. COVID-19 infection results in significant elevations in IL-6, ferritin and D-dimer, which are effectively associated with disease severity and progression [13]. Ruscitti et al. described the role of the heavy chains of ferritin (FeH) in activating macrophages (macrophage-activating syndrome; MAS), which stimulate the secretion of inflammatory cytokines, and thus cause massive cytokine storms in severe COVID-19 cases [14,15]. IL-6 induces the synthesis of many proteins, or can reduce their degradation. These include higher procalcitonin (PCT) levels in the presence of pro-inflammatory cytokines, probably due to the inhibition of PCT proteolysis [16]. All in all, it seems that IL 11R, ferritin and procalcitonin dysregulation are promising biomarkers of COVID infection.

Following viral RNA replication and the synthesis of its structural protein, the inflammatory cascade is activated by inflammatory sensors [17]. The cytokine storm is common in severe to critical cases of COVID-19, along with concomitant reductions in natural killer cells (NK) and lymphocyte cell counts, and increases in levels of C-reactive protein (CRP), D-dimer, procalcitonin and ferritin [18].

Moreover, SARS-CoV-2 components (proteins/nucleic acid) interact with the host’s biomolecules in a phenomenon known as host–pathogen interaction. One of the major actions of these long, highly mutated RNA viruses is their intrusion into the host’s coding and non-coding RNAs (RNA-related cellular processes), which plays a major role in viral pathogenesis and severity, affecting the host’s response to different stages of the infection [19,20].

LncRNAs [21] and miRNAs [22] have been identified as prognostic markers in viral infections, including COVID-19. For instance, miR-200c-3p was found to be activated during SARS infection, resulting in the downregulation of ACE2 [23]. Moreover, Sheng et al. [24] have studied the effects of different expressions of 20 miRNAs on COVID-19 patients and healthy controls, and concluded that these miRNAs are potential biomarkers for COVID-19. Recently, Farr et al. revealed three circulating miRNAs as a diagnostic signature useful in classifying patients in early stages, with the ability to discriminate COVID-19 and H1N1 infections from healthy controls [25]. On the other hand, MALAT1 LncRNA [26] and ANRIL LncRNA [27] have been identified to play roles in the induction of the inflammatory response, along with cytokine release. It has also been reported that the main causes of inflammatory cytokine storm in COVID-19 patients are interleukin-6 (IL-6) and the NOD-like receptor protein 3 (NLRP3) inflammasome [28]. Recently, Byron et al. [29] computationally determined that there are 22 Lnc-RNAs acting on the overexpression of 10 cytokines involved in the cytokine storm during COVID-19 infection. The authors recommend the utilization of these LncRNAs as assorted diagnostic/prognostic non-invasive biomarkers for the prediction of infection severity and phenotype.

The early prediction of severe and critical COVID-19 cases is a challenging diagnostic demand, but it would markedly enhance the available treatment plan/protocol, and provide subsequent improvements in infection control and disease management [9].

In this pilot study, we aimed to build an integrated, genetically linked [*mRNA-miRNA-lncRNA*] RNA panel based on extensive in silico analysis using various databases, such as KEGG, Gene cards, Gene ontology, miRWalk 3.0, MiRBase, mirDB, Target scan and RNAup Web server tool, to confirm the COVID-19 specificity of the selected IL11RA mRNA and its related non-coding RNAs, and of miRNA, with cytokine–cytokine receptor interaction. The main aim of the study is to a provide simple, sensitive and specific panel that will enhance our capacity for the early predication of severe cases of COVID-19, and thus improve the treatment protocol. Clinical validation of the whole panel in the context of COVID-19 patients and healthy controls has been performed. To the best of our knowledge, we were the first to develop a complete genetic network related to COVID-19 infection severity prediction.

## 2. Materials and Methods

### 2.1. Study Population

The current study was approved by the Ain Shams ethical committee, Faculty of Medicine, Cairo, Egypt. All the participating COVID-19 cases were admitted to the pulmonology department of Ain Shams University Hospital in 2020. The classification of SARS-CoV-2 severity was performed according to the Egyptian Ministry of Health (MOH) protocol version 1.4 [30] into mild, moderate and severe groups. There were 100 study subjects. In total, 59 were mild cases and 41 were severe cases, and there were 100 healthy volunteers who came for routine checkups at the hospital clinics (Pediatric, Chest and geriatric departments, Ain Shams University hospitals) and were confirmed by negative PCR results for SARS-CoV-2. All the study subjects were informed about the study, and written informed consent was received.

COVID-19 was diagnosed based on the clinical presentation along with qRT-PCR and routine laboratory investigations, including those for CBC, CRP, LDH, D-dimer, ferritin and procalcitonin. CT chest examinations were also performed.

Blood samples were collected upon hospital admission. The samples were processed by centrifugation at 4000 rpm for 20 min. The resulting sera were kept in aliquots at −80 °C in a freezer for further processing.

### 2.2. Total RNA Extraction and Quantitative Real-Time PCR (RT-qPCR)

Using a miRNEasy extraction kit (Qiagen, Hilden, Germany), total RNA was extracted and purified from the serum samples according to the manual. Further assessments of RNA concentration and purity were performed using the Qubit^TM^ ds DNA HS Assay Kit (catalogue number Q32851) and the Qubit ^TM^ RNA HS Assay Kit (catalogue number Q32852) (Invitrogen by Thermo Fisher Scientific, Eugene, OR, USA) with Qubit 3.0 Fluorimeter (Invitrogen by life technologies, Kuala Lumpur, Malaysia).

Reverse transcription was performed using equivalent amounts of RNA with a TaqMan^TM^ MicroRNA Reverse Transcription Kit (catalogue number 4366596) (Applied biosystems by Thermo Fisher Scientific, Balitics, UAB, made in Lithuania) and a High-Capacity cDNA Reverse Transcription Kit with RNAse Inhibitor (catalogue number 4374966, Applied Biosystems by Thermo Fisher Scientific, Carlsbad, CA, USA), using ProFlex^TM^ Base (Applied biosystems by Thermo Fisher Scientific, Woodlands, Singapore).

An IL11RA TaqMan probe and miR-4257 TaqMan probe with universal TaqMan master mix were used, and U6 was used as an endogenous reference.

The assessment of LncRNA RP11-773H22.4 was performed using the miScript II RT Kit (Qiagen, Hilden, Germany) to form cDNA; then, RT2 SYBR Green ROX qPCR Master Mix (Qiagen, Germany) was applied, and endogenous reference samples of ACTB-1 were assessed in duplicates. The relative quantification of expression was calculated via RQ = 2^−ΔΔCt^ using the Livak method [31], using real-time PCR, a 7500 Fast System (Applied Biosystems, Foster City, CA, USA) and a data analyzer. C_t_ values over 36 were interpreted as negative expression.

### 2.3. IL11RA and Procalcitonin Protein Quantification

A Human Interleukin 11 RA ELISA kit (Invitrogen, Thermo Fisher Scientific, Frederick, MD, USA) was used to measure the level of IL11RA protein in a patient’s serum, within the reference range of 3.29–800 pg/mL.

Additionally, a Human Procalcitonin ELISA kit (Invitrogen, Thermo Fisher Scientific, Carlsbad, CA, USA) was used to measure the level of procalcitonin in a patient’s serum, within a reference range of 27.43–20,000 pg/mL. Both proteins were assessed with a Varioskan microplate reader (Thermo Fisher Scientific, Woodlands, Singapore).

### 2.4. Statistical Analysis of Results

The software package of statistical analysis version number 25 (SPSS25, IBM, Illinois, Chi, USA) was used to statistically analyze the output data. Quantitative variables were analyzed using the median and mean ranks for the non-parametric data, and the mean ± SD were used for the symmetrically distributed raw numerical data. One-way ANOVAs, cross-tabulation and Mann–Whitney test were also used as appropriate. Qualitative variables were evaluated using chi-square tests for number and percentage calculations. Correlations between quantitative variables were assessed using Spearman correlation tests; correlation coefficients were estimated. To evaluate the predictive value of the RNA panel as regards COVID-19, the receiver operating characteristic (ROC) curve was used, in order to estimate the best cut-off points for different parameters with optimal sensitivity and specificity. To determine the predictive power of different biomarkers for SARS-CoV-2 severity, we performed a multivariable logistic regression analysis. A *p*-value < 0.05 was considered significant.

## 3. Results

### 3.1. Bioinformatics and Dataset Analysis

We first reviewed the currently available literature on the pathophysiology and molecular signaling pathways involved in the cytokine storm related to the pathogenesis and severity of COVID-19, focusing on the Kyoto Encyclopedia of Genes and Genomes (KEGG) map pathways database, and specifically on cytokine-related pathways. Of note, interleukin-6 (IL-6) is proposed to be associated with the severity of COVID-19 [32]. 

Then, pursuing our interest in the cytokine signaling pathway, we assessed IL11RA, as previous studies have asserted that cytokines IL-11 and IL-6 stimulate intracellular signaling events through a homodimer of β-receptor glycoprotein 130 [10,12].

Thus, IL11RA mRNA was first selected based on its strong correlation with IL-6, its novelty and its basal expression in the peripheral blood (Supplementary Figures S1 and S4). Secondly, higher ferritin levels related to secondary cytokine storm syndrome have been reported in severe COVID-19 patients due to secondary hemophagocytic lymphohistiocytosis. Increased ferritin is a central characteristic of cytokine syndromes, COVID-19 severity and poor prognosis [13,33]. Yesupatham and colleges assessed interleukin-6 and ferritin levels, and their clinical correlations, among COVID-19 patients.

Thirdly, Sharma and his colleagues [34] found interesting correlations between interleukin expression and sepsis in neonates, which strengthens the literature on this topic.

Our results were confirmed via the NCBI and Gene cards databases. Gene ontology was performed to ensure the link of the genes to cytokine response, using the online database, Enrichr (http://amp.pharm.mssm.edu/Enrichr) (accessed 1 November 2021) (Supplementary Figure S2) [35,36]. Using the Enrichr software, three integrated genes were mapped into the PPI network, providing more evidence of their putative interactions. In general, IL 11R, ferritin and procalcitonin dysregulation may serve as promising biomarkers of COVID infection based on both the literature search/review and in silico data analyses.

Interactions among miRNAs and the selected mRNAs were predicted using the available online databases miRWalk 3.0 (http://mirwalk.umm.uni-heidelberg.de/) (accessed 1 November 2021), which integrated the prediction results of both TargetScan (v7.0; targetscan.org) [37] and MiRBase (accessed online 1 November 2021) [38]. Fourthly, the mirDB database was used for retrieving miR-4257(Accession number: MIMAT0016878), using the same criteria regarding expression in peripheral blood, novelty and high numbers of complementarily binding sites (Supplementary Figures S4, S5 and S7). Confirmatory pathway enrichment analysis was performed for the selected miRNA to ensure its relations to cytokines. A target scan confirmed the predicted consequential pairing between miRNA-4257 (ENSG00000264553) and IL11RA mRNA (ENSG00000137070) (raw 3’ pairing score <3.0) at position 170–177 of IL11RA 3’ UTR (Supplementary Figures S8 and S9). Then, using the miRWalk database, we selected LncRNA RP11-773H22.4 (ENST00000588211.1) as the controller of the selected genes, based on its complementary alignment with miRNA-4257 and IL11RA mRNA. Sequence alignment was performed among LncRNA RP11-773H22.4, miR-4257 and IL11RA mRNA (Supplementary Figures S10–S12)).

Moreover, the enricher database analysis of the retrieved genetic network returned interactions with different cell cycle cytokines and pro-inflammatory proteins, such as, but not limited to, EOMES, which is essential for the T-cell mediated immune response against pathogens; and also the depending of Ras-MAPK activation on E2F (Supplementary Figure S2).

Finally, we assessed the thermodynamic interaction of LncRNA–miRNA binding, using the online database (accessed 1 November 2021) RNAup web server tool in Vienna RNA web server: (http://rna.tbi.univie.ac.at/ (accessed 1 November 2021) to determine whether the interaction between them is thermodynamically favorable or not (Supplementary Figures S13 and S14)

### 3.2. Clinical and Biochemical Indices

Sex, age and serum hemoglobin level showed no significant differences between the COVID-19 group and the healthy control group (*p* > 0.05). On the contrary, there were significant differences between the studied groups concerning total leukocyte count (TLC; *p* = 0.002), lymphocyte count (*p* = 0.000), platelet count (*p* = 0.006), C-reactive protein serum level (*p* = 0.000), LDH serum level (*p* = 0.000) and *D*-dimer serum level (*p* = 0.000), as shown in Table 1.

### 3.3. Differential Expression of the Severity Predictors in the Investigated Groups

**Figure 1:**
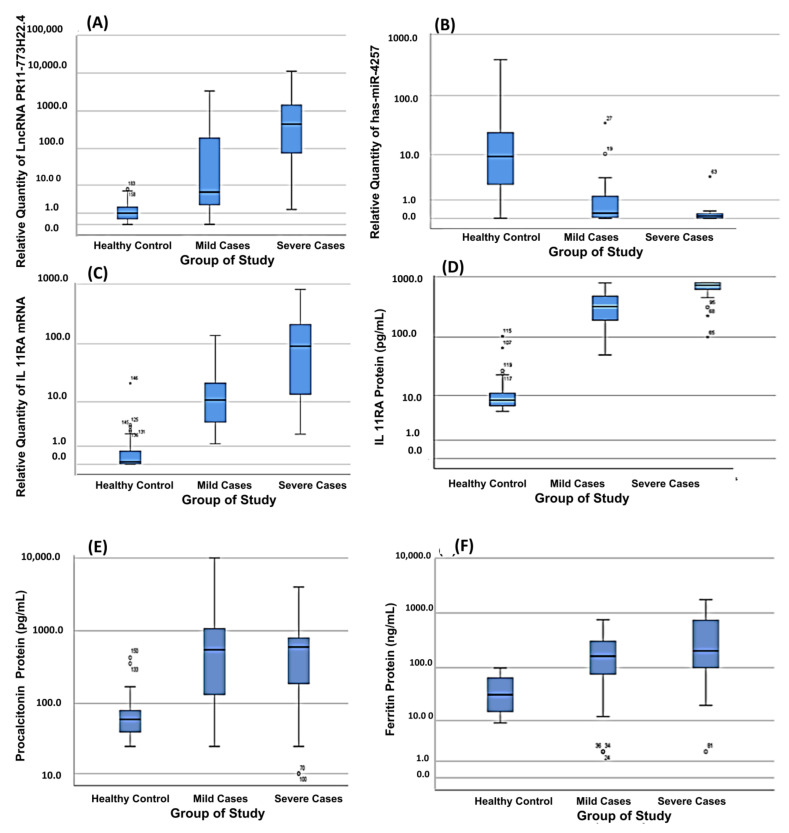
The differential box plot analysis of the investigated biomarkers, measured by qRT-PCR or ELISA, among the study groups. (**A**) LncRNA RP11-773H22.4, (**B**) HSA-MIR-4257, (**C**) IL11RA mRNA, (**D**) IL11RA protein, (**E**) pro-calcitonine and (**F**) ferritin. The median is represented as a line inside the box, and the 1st and 3rd quartiles are represented by the top and bottom lines of the box, respectively. Dots represent outliers.

The expression of the IL11RA molecular network was assessed via the fold-change (RQ) values in the different investigated groups (mild COVID-19, severe COVID-19 and healthy control) in order to confirm the retrieved bioinformatics data. As expected, IL11RA mRNA and LncRNA RP11-773H22.4 expressions were upregulated, and hsa-miR-4257 was downregulated in the COVID-19 group compared to the healthy control group (*p* < 0.000) (Figure 1A–C). Interestingly, the expression pattern of the IL11RA molecular network was discriminative between COVID-19-positive patients and healthy controls, and between mild and severe COVID-19 compared to healthy controls.

IL11RA mRNA and LncRNA RP11-773H22.4 were overexpressed by 100-fold and 6-fold in mild COVID-19 cases compared to the healthy control group, and by 9-fold and 72-fold in severe COVID-19 cases compared to mild COVID-19 cases, respectively (Figure 1A–C). Additionally, hsa-miR-4257 was discriminatory between COVID-19-positive patients and healthy controls; mild cases and healthy controls; and mild and severe cases of COVID-19, compared to healthy controls. The expression of hsa-miR-4257 was downregulated 35-fold in mild COVID-19 cases compared to the healthy control group, and 3-fold in severe COVID-19 cases compared to mild cases, as shown in Table 2 and Figure 1A–C.

Moreover, by assessing the serum levels of ferritin, procalcitonin (ENSGOOOOO110680) and IL11RA proteins (ENST00000441545.7) in the studied groups, we found significant increases in the three proteins in the COVID-19 group compared to the healthy control group (*p* < 0.000) (Table 2, Figure 1D–F). These increases in the IL11RA, ferritin and procalcitonin are in agreement with other findings, as ferritin and procalcitonin have been used as prognostic biomarkers for determining disease severity [39,40,41]. However, based on the obtained results, procalcitonin cannot discriminate among mild and severe cases.

**Figure 2:**
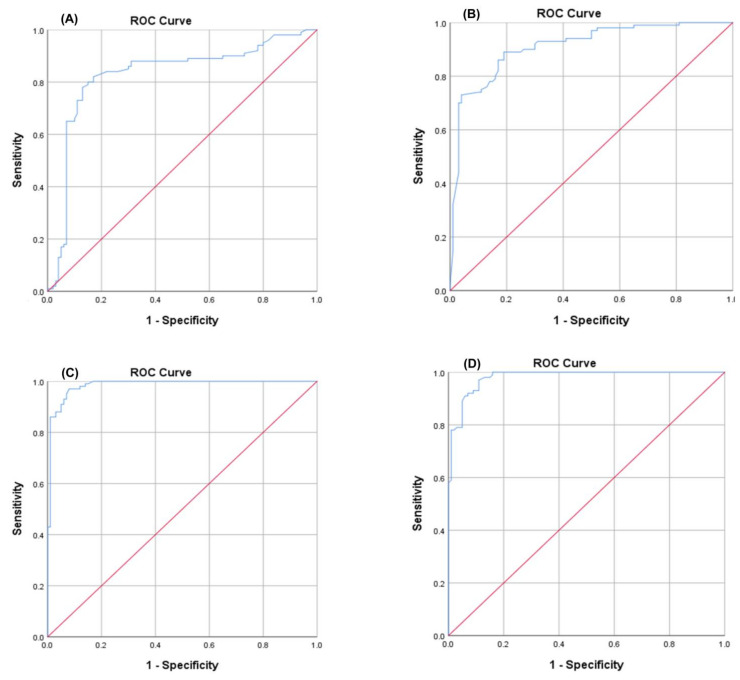
The receiver operator characteristic (ROC) curve presents the diagnostic accuracy of the IL11RA mRNA panel used to discriminate between SARS-CoV-2 and the control. (**A**) LncRNA RP11-773H22.4, (**B**) HSA-miRNA-4257, (**C**) IL11RA mRNA and (**D**) IL11RA protein.

ROC curve analysis was performed for the investigated COVID-19 patients versus the healthy control group to determine the best cutoff value. The ideal cutoff values were 1.15 for IL11RA mRNA (AUC = 0.985), 2.25 for LncRNA RP11-773H22.4 (AUC = 0.829) and 2.07 for hsa-miR-4257 (AUC = 0.911) (Figure 2). The estimated sensitivities were 100%, 86.2% and 88%, respectively; the IL11RA protein showed 100% sensitivity and an AUC = 0.995%, which validates the genetic network data. Consequently, the retrieved genetic network can be used for discrimination between COVID-19 patients and healthy individuals (Table 3, Figure 2).

**Figure 3:**
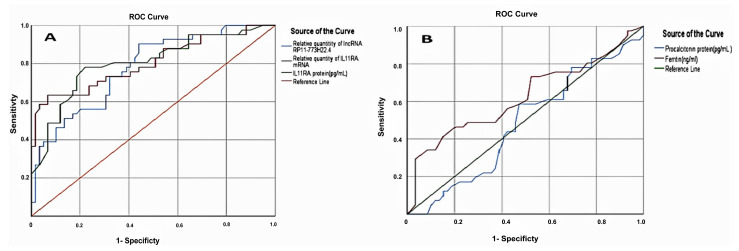
The receiver operator characteristic (ROC) curve presents the diagnostic accuracy of the IL11RA mRNA panel discriminating between mild and severe cases of SARS-CoV-2: (**A**) IL11RA mRNA and protein, and LncRNA RP11-773H22.4. (**B**) Procalcitonin and ferritin.

The routine COVID-19 serum markers (ferritin and procalcitonin), along with our retrieved novel marker, the IL11RA protein, have been analyzed using the ROC curves of the investigated groups. The ideal cutoff values were 77 for ferritin (AUC = 0.869), 174 for procalcitonin (AUC = 0.807) and 42 for IL11RA (AUC = 0.981) (Figure 3). Accordingly, these cutoff values could be used to differentiate between COVID-19 cases and healthy controls, with sensitivity values of 74%, 70% and 100%, respectively (Table 3, Figure 3).

The use of the selected LncRNA and IL11RA mRNA in assessing the severity of infection was made efficient using ROC curve analysis, with the ideal cutoff values of 15.95 for IL11RA mRNA (AUC = 0.803) and 40.5 for LncRNA RP11-773H22.4 (AUC = 0.777). These can be used to differentiate mild from severe COVID-19 cases, with sensitivities of 73.2% and 78% and specificity values of 76% and 71%, respectively (Table 4, Figure 3). Additionally, the best cutoff values were 146 for ferritin (AUC = 0.624), 447 for procalcitonin (AUC = 0.480) and 425 for IL11RA protein (AUC = 0.803), with sensitivities of 61%, 61% and 80.5%, respectively (Figure 3).

**Figure 4:**
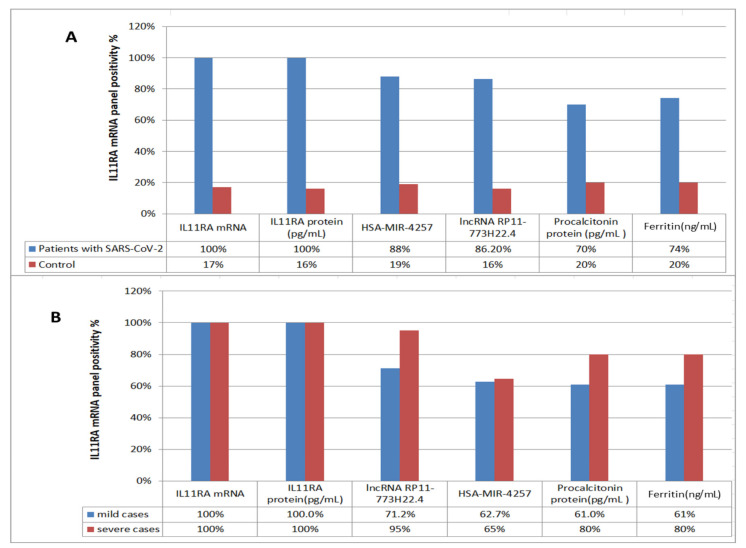
Bar chart showing the positivity rates of the studied parameters: (**A**) between SARS-CoV-2 patients and control, and (**B**) the diagnostic value of the studied parameters in discriminating mild from severe patients with SARS-CoV-2.

These data support the other data showing that discrimination between mild and severe COVID-19 cases could be achieved at the protein level (Table 3, Figure 3). Moreover, the best cutoff values were 15.95 for IL11RA mRNA (AUC = 0.803) and 40.5 for LncRNA RP11-773H22.4 (AUC = 0.777), and these could be used to differentiate mild from severe COVID-19 cases, with sensitivities of 73.2% and 78% and specificity values of 76% and 71%, respectively (Table 3, Figure 4). Thus, the selected RNA panel was superior to other clinical parameters, such as ferritin and procalcitonin. Indeed, identifying patients at risk of severe COVID-19 infection will help clinicians to plan the most appropriate early management approach for each patient.

The above two ROC curve analyses (RNA level and protein level) show that the IL11RA mRNA and its protein have the best cutoff values and AUCs for discriminating between mild and severe cases of SARS-COV-2 (Figure 3). Moreover, lncRNA RP11-773H22.4 and hsa-miR-4257 showed better sensitivities (86.2% and 88%) than ferritin and procalcitonin (74% and 70%, respectively). These results suggest that the selected RNA panel could be used as a tool to differentiate mild from severe COVID-positive cases.

In the same context, the pattern of increases in ferritin, procalcitonin and IL11RA was highly discriminative between mild and severe cases of COVID-19, compared to healthy controls. On the other hand, the elevation of ferritin, procalcitonin and IL11RA by 5-fold, 9-fold and 36-fold in mild COVID-19 cases compared to the healthy control group, and by 1.2-fold, 1-fold and 2-fold in severe COVID-19 cases compared to mild COVID-19 cases, respectively, confirmed that the selected protein panel could be used in COVID-19 severity discrimination (Figure 4).

Regarding the severity of COVID-19, by using univariate analysis, it was determined that LncRNA RP11-773H22.4 (*p* = 0.04) is an independent prognostic factor besides age (*p* = 0.001), co-morbidities (*p* = 0.032), ventilation, the case severity and CT chest findings (*p* = 0.000), as shown in Table 4.

Additionally, the multivariate analysis revealed that LncRNA RP11-773H22.4, hsa-miR-4257, IL11RA mRNA, and IL11RA protein levels were independent prognostic factors besides the serum ferritin level and CT findings, encouraging the use of novel COVID-19 severity predictors along with the more routine ones (*p* ≤ 0.05), as shown in Table 5.

### 3.4. Correlations among the IL11RA Molecular Network and Protein Predictors (Ferritin, Procalcitonin and IL11RA)

**Figure 5:**
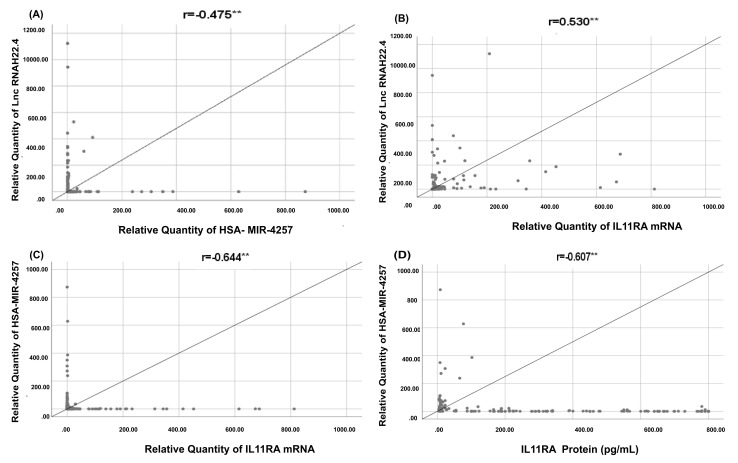
Correlations among IL11RA molecular network and IL11RA protein. As shown each two parameters are correlated together (**A**) the relative quantity of has-miR-4257 and Ln RNA H22.4, showing increase in the Long nc RNA compared to the miRNA negative correlation, (**B**) The correlation between the IL-11R mRNA and the Lnc RNA H22.4, showing that positive correlation between the two variables, (**C**) The correlation between the IL-11R mRNA and has-miR-4257, showing negative correlation and finally (**D**) the correlation between IL 11RA protein and the has-miR-4257, which shows negative correlation and so as the IL-11RA increase the has-miR-4257 decrease. ** are Statistically significant.

There was strong positive correlation among lncRNA RP11-773H22.4, ferritin, procalcitonin, IL11RA protein and IL11RA mRNA (*p* = 0.000). Contrarily, there was a strong negative correlation between hsa-miR-4257 and IL11RA mRNA (*p* = 0.000) (Supplementary Table S1, Figure 5). SARS-CoV-2 infection results in the upregulation of LncRNA RP11-773H22.4, which leads to the downregulation of hsa-miR-4257 and the subsequent upregulation of IL11RA mRNA. These data validate our bioinformatics findings and highlight the role of the novel molecular network in COVID-19 disease and the prediction of its severity.

## 4. Discussion

The COVID-19 pandemic is a serious global problem for many reasons, the most important of which being that it causes severe acute respiratory syndrome. Consequently, the infection can cause critical, life-threatening respiratory injuries, for which there is no specific therapeutic treatment available to date [32].

The SARS-CoV-2 pandemic is not just a health problem—social and economic dimensions have become involved too, which have affected the entire globe over the last two years.

As regards the constant and significant mutations taking place in the virus’s whole genome, and specifically in the spike protein (enabling it to escape the immune system and thus increase infectivity, leading to serious novel symptoms/complications [42]), there is a strong demand for a robust, sensitive, rapid and specific diagnostic tool/assay that can evaluate the severity of the infection.

Despite the presence of many serum-based laboratory tests, antibody detection serological tests and many real-time PCR-based assays, most still have drawbacks. Moreover, there is great variability in the sensitivity and specificity of existing PCR-based tests, depending on the vendor, the strain, the viral load and the location at which the swab is used [43,44]. Additionally, PCR results should be correlated with patient history and symptoms for infection confirmation; for instance, a negative COVID-19 PCR test does not exclude infection for the above-mentioned reasons. Thus, negative PCR results should be confirmed through clinical observation, chest CT scans and other diagnostic serum markers, to reach the best clinical decision concerning infection, severity and treatment protocol [45].

The severity of COVID-19 infection is critical, affecting disease mortality and spread rate [45]. Thus, developing novel, and non-invasive biomarkers, will empower the early detection and infection severity; leading to control the virus spreading, and enhance treatment management. Therefore, research should be interested not only in therapy and vaccine development, but also in developing markers for COVID-19 severity prediction [46].

Zeng et al. found a higher concentration of SARS-CoV-2 IgG in females than in males in severe cases, and this could be a protective mechanism in females [46]. In another study by Shen et al. [47], severe cases that received convalescent plasma with a high titer of COVID-19 antibodies recovered, and despite the low number of subjects in this study, this result, along with those of other studies, suggests that detecting COVID-19 severity as early as possible will lead to significantly better disease management and potential treatment outcomes [46].

The non-coding RNAs ((Lnc-RNAs), miRNAs, Piwi RNAs and others) play a crucial regulatory role in the cell at various levels, such as in epigenetics, structure–structure (RNA, DNA and proteins) interactions and interactions of complementary sequences, in addition to cancer progression and the inhibition or activation of infections [48,49,50].

Long non-coding RNAs (Lnc-RNAs) are a group of cellular RNAs that are more than 200 nucleotides in length. Increasing amounts of evidence suggest that both Lnc-RNAs and miRNAs play vital roles in the pathogenesis of different diseases. Much literature has reported the strong relation between viral infections and Lnc-RNAs in different ways [51,52].

In the same context, there is increasing evidence that miRNAs play major roles in the pathogenesis, diagnostics and treatment of viral infections, such as human immunodeficiency virus 1 (HIV-1) [53,54,55], hepatitis C (HCV) [56,57] and herpes simplex viruses (HSV) [58,59,60]. For instance, miR-122 is a main component of HCV, with complementary sequences in its 5′UTR. The presence of the virus depends mainly on the replication of miR-122; by knocking down miRNA-122, HCV replication is reduced, and the virus is limited. An anti-sense targeting of miR-122 is in the clinical trial stage as a treatment for HCV [61]. Additionally, miRNA 332 and miRNA 628 clearly interact with the MERS-CoV viral genome [62]. Based on the importance of miRNAs, it is essential to recognize the miRNAs regulating and interacting with COVID-19 disease [63]. As miRNAs have binding sites for both mRNAs and Lnc-RNAs, the latter can act as a competitive endogenous RNA (ceRNA) to suppress the miRNA function, or as a precursor and encoder of miRNAs [64].

Furthermore, miRNAs/Lnc-RNAs play a vital role in the relation between host and virus, mainly in regulating the transcription of virus and host genes [65]. Cheng et al. reported the upregulation of ENSG00000231412 and ENSG00000274173 LncRNA in severe cases of COVID-19 [66].

Regarding IL-11, its upregulation and overexpression in the lungs is usually linked with viral infections, including SARS-CoV-2, and IL-11 induces lung fibrosis and epithelial dysfunction [67]. Hence, IL-11 RA is overexpressed to mediate the cascade of IL-11 function, as discussed below [68] (Figure 1), Table 1.

Interleukin 11 (IL11) is usually compared to interleukin 6 (IL6), both of them begin signaling by making hexameric complexes with their receptors IL11RA, IL6R and Glycoprotein 130 (gp130) receptor, respectively [69].IL11 is a multifunctional cytokine derived from stromal cells, and is a member of the gp130 family. It was first isolated from a bone marrow-derived cell line [70]. The signaling mechanism initiated by IL-11 is mediated by its receptor, IL11RA, which uses common subunits of the gp130 receptor, such as IL-6, oncostatin M, leukemia inhibitory factor and ciliary neurotrophic factor [71].IL11RA requires the co-expression of a common subunit of the gp130 receptor for signal transduction [72]. The main pathways activated upon IL-11 stimulation are Ras-MAPK, JAK-STAT, PI3K-AKT and NF-kappa B [73].

In addition to the pathways that act synergistically with IL6 via their hexameric complexes [74], two isoforms of IL11RA that differ in their cytoplasmic domains have been recognized [75]. Nakayama et al. reported high levels of IL11RA mRNA in gastric cancer [76]. Lay et al. found high levels of IL11RA in endometrial cancer [77]. It is expected that the expression of IL11 will increase during COVID-19 infection, as it is a pro-inflammatory cytokine that regulates platelet maturation and causes bone resorption along with IL6 [78,79], all of which comprise the clinical picture of COVID-19 infection.

Our in silico analysis, as described in the Methods section, Results and Supplementary Materials, revealed an IL11RA protein/IL11RA mRNA/miR-4257/ lncRNA RP11-773H22.4 molecular network that is highly determinative of COVID-19 severity and acts along with procalcitonin and ferritin (Figure 1 and Figure 2, Table 1). These supposed RNAs of the panel, together with IL-11, procalcitonin and ferritin, are formed in the liver and other inflammatory cells, and then released to the blood as inflammation associated markers [80].

To confirm and validate the in silico findings, this pilot study assessed the differentiation among normal, mild and severe cases in clinical subjects. There are three main novel findings. The first finding was the significant expression level of LncRNA RP11-773H22.4 in SARS-CoV-2 cases, with highly discriminative cutoff values that could be used in the differentiation between COVID-19 cases and healthy controls, and between severe and mild COVID-19 cases (Figure 1, Figure 2, Figure 3 and Figure 4). The second finding was the clear and significant downregulation of the has-miR-4257 expression level in COVID-19 cases, and vice versa in the healthy control group. A discriminative cutoff value was calculated for differentiating between COVID-19 cases and healthy controls, and between severe and mild cases of COVID-19 (Figure 1, Figure 2, Figure 3 and Figure 4). The third finding was the upregulation and high level of expression of IL11RA mRNA, along with the IL11RA protein, in SARS-CoV-2. Discriminative cutoff values were identified for differentiating between COVID-19 cases and healthy controls, and between severe and mild cases (Figure 1, Figure 2, Figure 3 and Figure 4).

Thermodynamic assessment has been performed using the RNAup Web server (Vienna RNA Web server) to confirm the interaction between the long non-coding RNA and the miRNA retrieved in this study. It was found that, upon hybridization, the optimal secondary structure, as shown in (Supplementary Figures S13–S14), ranges from positions 218 to 223 in LncRNA, and from 57 to 62 in the miRNA.

The opening energy of LncRNA RP11-773H22.4 was computed as 3.76 kcal/mol, and that of has-miR-4257 was computed to be 2.21 kcal/mol. The total free energy for binding was −6.83 kcal/mol, and that for duplex formation was −12.80 kcal/mol. This means that the energy required for duplex formation (interaction between the two RNAs) is high (−12.80 kcal/mol), but their opening energy must be released in order to reach the total binding energy, which is 6.83 kcal/mol. As such, the energy for binding between the two RNAs is considered high. This can be explained mathematically by the addition of the two RNAs’ open energy values, and the addition of the duplex formation energy to the result: [LncRNA RP11-773H22.4 open energy] + [HSA-miR- 4257 open energy] *3.76* + *2.21* = *+5.9* [Total open energy of the two RNAs] + [Energy from duplex formation] *5.9* + (*−12.80*) = −6.83 kcal/mol. Thus, we can hypothesize that the SARS-CoV-2 virus induces the expression of LncRNA RP11-773H22.4, which acts as a sponge for miR-4257, leading to the upregulation of IL11RA mRNA expression, and then IL11 RA, followed by an increase in the cytokine response.

Along with the novel network identification, confirmation tests have been performed, and a significant relation has been found between our network and ferritin and procalcitonin, which are considered potent markers for COVID-19 diagnosis and severity determination. We also performed the other routine laboratory tests and chest CT scans (Table 1).

The genetic network retrieved in this study is related specifically to COVID-19 infection, and it has been utilized in the discrimination of infection severity. It could be employed as a non-invasive biomarker(s) for COVID-19 prognosis and diagnosis.

To the best of our knowledge, this is the first study to report the significant association between the differential expressions of the IL11RAprotein, IL11RA mRNA/miR-4257/lncRNA RP11-773H22.4 molecular network and SARA-CoV-2, in addition to its potential discriminative ability among healthy, mild and severe SARS-CoV-2 cases (disease severity).

## 5. Conclusions

The early prediction of COVID-19 disease severity in symptomatic and asymptomatic patients, using simple, specific and sensitive tools, will help in controlling the spread of the disease, and enhance its management. This is essential, along with developing vaccines, and genetic and serological diagnostic techniques. In this study, we have validated a non-invasive tool for the prediction of disease severity, distinguished among mild, severe and normal cases. We explored bioinformatics databases to retrieve a genetic network (mRNA–miRNA–LncRNA), and clinically validated it in different target groups. We finally concluded that the IL11RA molecular network could be used for predicting the severity of COVID-19 alone or in combination with the existing biomarkers procalcitonin and ferritin. This is also significant to viral pathogenesis exploration, as it helps identify more genetic networks and biomarkers related to COVID-19 pathogenesis.

## Figures and Tables

**Table 1:** The demographic, clinical and biochemical indices of the study groups.

	Cases N = 100			Control N = 100			Test of Significance
Sex Male: n = 108 (54%) Female: n = 92(46%)	56 (56%) 44 (44%)			52 (52%) 48 (48%)			X_2_ = 0.322 *p* = 0.57
Chest CT finding Ground glass opacity Single lung infiltrate Bilateral lung infiltrate	42(42%) 34(34%) 24(24%)			NA			NA
Co-morbidities +ve: n = 74(37%) −ve: n = 126 (63%)	56 (56%) 44 (44%)			18 (18%) 82 (82%)			X_2_ = 30.9 *p* = 0.000 *
Severity Mild Severe	59 (59%) 41(41%)			NA			NA
Ventilation +ve −ve	64 (64%) 36 (36%)			NA			NA
Outcome Recovery Recurrence Death	49 (49%) 19 (19%) 32 (32%)			NA			NA
	Mean	±SD	Standard error	Mean	±SD	Standard error	Test of Sig.
Age/years	32.795	19.9122	1.9912	34.275	21.6260	2.1626	F = 0.623 *p* = 0.43
TLC (thousands/cmm^3^)	16.7375	28.29100	2.82910	9.3620	11.59069	1.15907	F = 6.7 *p* = 0.002 *
Lymphocytes (×10^9^/L)	1.1871	0.53824	0.05382	3.2460	0.86672	0.08667	F = 19.17 *p* = 0.000 *
Hemoglobin (g/dL)	11.9730	2.18696	0.21870	12.2080	2.46518	0.24652	F = 0.017 *p* = 0.897
Platelets (thousands/cmm)	262.7300	84.96331	8.49633	244.5200	99.78001	9.97800	F = 7.6 *p* = 0.006 *
C-reactive protein (mg/L)	66.5030	75.35360	7.53536	0.8818	1.34212	0.13421	F = 129.74 *p* = 0.000 *
Lactate dehydrogenase (LDH)(U/L)	357.24500	244.410944	24.441094	166.52000	50.399311	5.039931	F = 160.056 *p* = 0.000 *
D-dimer (mg/L)	137.20964	442.970546	44.297055	0.12720	0.108172	0.010817	F = 31.058 *p* = 0.000 *

F: One-way ANOVAs test, X_2_: chi-square test; TLC: total leukocyte count; co-morbidities, including diabetes mellitus, hypertension, asthma and combined co-morbidities. *: statistically significant.

**Table 2:** The differential expression of various parameters is shown among the different study groups, in addition to severe versus mild cases.

		Median	Mean Rank	Chi-Square	*p*
LncRNA RP11-773H22.4	Healthy control	1.1000	67.60	^a^ 78.9	^a^ 0.000
	Mild	6.2000	115.19	22.050 ^b^	^b^ 0.978
	Severe	448.0000	159.62		^c^ 0.001
HSA-MIR-4257	Healthy control	10.5050	141.61	^a^ 106.8	^a^ 0.001
	Mild	0.3000	70.58	^b^ 9.416	^b^ 0.002
	Severe	0.1000	43.29		^c^ 0.012
IL11RA mRNA	Healthy control	0.1000	51.9	^a^ 148.07	^a^ 0.000
	Mild	10.7000	135.5	^b^ 26.4	^b^ 0.578
	Severe	91.3910	176.7		^c^ 0.000
IL11RA protein (pg/mL)	Healthy control	8.9000	52.37	^a^ 146	^a^ 0.000
	Mild	321.0000	135.30	^b^ 26.4	^b^ 0.00
	Severe	667.0000	167.83		^c^ 0.000
Procalcitonin protein (pg/mL)	Healthy control	60.0000	69.78	^a^ 65.5	^a^ 0.000
	Mild	550.0000	133.09	^b^ 0.118	^b^ 0.00
	Severe	600.0000	128.54		^c^ 0.731
Ferritin (ng/mL)	Healthy control	31.5000	63.58	^a^ 83.16	^a^ 0.000
	Mild	162.3000	131.09	^b^ 4.429	^b^ 0.00
	Severe	203.0000	146.52		^c^ 0.035

^a^ Statistics among all groups. ^b^ Statistics: mild versus healthy control; ^c^ mild versus severe cases; *p*-value > 0.05 is considered statistically non-significant; and *p*-value < 0.05 is considered statistically significant. F: one-way ANOVA.

**Table 3:** The performance characteristics of the different investigated laboratory parameters.

**Performance Characteristics in SARS-CoV-2 Patients Versus Healthy Control**								
Test Result Variables	Area under Curve	Std. Error	*p*	Asymptotic 95% Confidence Interval		Cutoff	Sensitivity	Specificity
				Lower Bound	Upper Bound			
Procalcitonin protein (pg/mL)	0.807	0.032	0.000	0.744	0.871	174	70%	80%
Ferritin (ng/mL)	0.869	0.027	0.000	0.817	0.922	77	74%	80%
IL11RA mRNA	0.985	0.007	0.000	0.972	0.999	1.15	100%	83%
IL11RA protein (pg/mL)	0.981	0.007	0.000	0.967	0.995	42	100%	84%
HSA-MIR-4257	0.911	0.021	0.000	0.871	0.951	2.07	88%	81%
LncRNA RP11-773H22.4	0.829	0.032	0.000	0.766	0.892	2.25	86.2%	84%
**Performance Characteristics in Mild Versus Severe SARS-CoV-2 Patients**								
Procalcitonin protein (pg/mL)	0.480	0.059	0.731	0.365	0.594	447	61%	41%
Ferritin (ng/mL)	0.624	0.060	0.036	0.507	0.741	146	61%	50%
LncRNA RP11-773H22.4	0.777	0.046	0.000	0.687	0.867	40.5	78%	71%
IL11RA mRNA	0.803	0.047	0.000	0.710	0.895	15.95	73.2%	76%
IL11RA protein (pg/mL)	0.803	0.046	0.000	0.712	0.894	425	80.5%	76%

**Table 4:** Predictors’ outcomes regarding COVID-19 severity in infected patients.

	Recovery N = 49		Recurrence N = 19		Death N = 32		Test of Significance
Age/years	Mean 25.235	SD 18.7053	Mean 38.053	SD 16.6616	Mean 41.250	SD 19.5498	KWχ2 = 809 *p* = 0.001 *
Sex Male: n = 56 (56%) Female: n = 44 (44%)	28 (51.7%) 21 (42.9%)		12 (63.2%) 7 (36.8%)		16 (50%) 16 (50%)		X_2_ = 0.889 *p* = 0.641
Co-morbidities +ve 44 (44%) −ve 56 (56%)	26 (53.1%) 23 (46.9.7%)		10 (52.6%) 9 (47.4%)		8 (25%) 24 (75%)		X_2_ = 6.896 *p* = 0.032 *
Ventilation +ve 36 (36%) −ve 64 (64%)	2 (4.1%) 47 (95.9%)		4 (21.1%) 15 (78.9%)		30 (93.7%) 2 (6.3%)		X_2_ = 69.9 *p* = 0.000 *
Severity Mild (n = 59) Severe (n = 41)	46 (93.3%) 3 (6.1%)		13 (68.4%) 6 (31.6%)		0 (0%) 32 (100%)		X_2_ = 71.378 *p* = 0.000 *
Chest CT finding Ground glass opacity Single lung infiltrate Bilateral lung infiltrate	29 (59.2%) 18 (36.7%) 2 (4.1%)		13 (68.4%) 1 (5.3%) 5 (26.3%)		0 (0%) 15 (46.9%) 17 (53.1%)		X_2_ = 45.778 *p* = 0.000 *
	Mean	SD	Mean	SD	Mean	SD	
Hemoglobin (gm/dL)	12.2510	2.36556	12.2158	1.75159	11.4031	2.08195	KWχ2 = 1.6 *p* = 0.223
Total leukocyte count (TLC) (thousands/cmm)	18.6347	31.86161	10.4316	4.04352	17.5766	30.72309	KWχ2 = 591 *p* = 0.556
Platelets (thousands/cmm)	253.6327	96.43169	260.8421	59.18076	277.7813	79.08498	KWχ2 = 0.784 *p* = 0.459
Lymphocytes (×10^9^/L)	1.2435	0.50182	1.1395	0.50617	1.1291	0.61368	KWχ2 = 0.542 *p* = 0.594
C-reactive protein (mg/L)	63.6531	77.46586	84.2947	88.52500	60.3031	63.59897	KWχ2 = 0.668 *p* = 0.515
Serum LDH (U/L)	344.74490	275.867800	310.73684	216.314288	300.00000	231.899867	KWχ2 = 0.337 *p* = 0.715
D-dimer (mg/L)	53.03259	267.717708	31.48211	64.400389	328.88147	676.230333	KWχ2 = 4.7 *p* = 0.01 *
Ferritin (ng/mL)	275.4490	363.82781	354.3684	424.74139	413.9188	412.48398	KWχ2 = 0.308 *p* = 0.735
Procalcitonin	1152.4694	1628.97473	1031.9474	2225.97301	865.5313	1050.06679	KWχ2 = 1.24 *p* = 0.294
*IL11RA mRNA* Positive (100%) Negative (0%)	49 (100%) 0 (0%)		19 (100%) 0 (0%)		32 (100%) 0 (0%)		NA
*IL11RA protein* (pg/mL) Positive (100%) Negative (0%)	49 (100%) 0 (0%)		19 (100%) 0 (0%)		32 (100%) 0 (0%)		NA
HAS-MIR-4257 Positive (88%) Negative (12%)	43 (87.8%) 6 (12.2%)		15 (78.9%) 4 (21.1%)		30 (93.8%) 2 (6.3%)		X_2_ = 2.479 *p* = 0.29
LncRNA RP11-773H22.4 Positive (81%) Negative (19%)	35 (71.4%) 14 (28.6.2%)		16 (84.2%) 3 (15.8%)		30 (93.8%) 2 (6.3%)		X_2_ = 6.424 *p* = 0.04 *

X_2_: Chi-square test; KWχ2: Kruskal–Wallis test; * statistically significant.

**Table 5:** Multivariate analysis using the existing and the proposed predictors of SARS-CoV-2 severity.

Variable	Score	Degree of Freedom	Significance	B	S.E.	Exp(B)
CRP	0.512	1	0.474	0.088	34.487	1.092
Hb	8.936	1	0.003 *	1.862	522.178	6.440
TLC	0.001	1	0.976	−0.171	138.882	0.843
Lymphocytes	3.825	1	0.051	−3.762	2063.186	0.023
LDH	0.588	1	0.443	−0.091	10.364	0.913
PLT	0.725	1	0.394	0.041	11.637	1.042
Co-morbidities	16.604	1	0.000 *	2.245	1006.505	9.441
Relative quantity of LncRNA RP11-773H22.4	11.286	1	0.001 *	−0.006	1.071	0.994
Relative quantity of HSA-MIR-4257	5.178	1	0.023 *	5.602	3992.203	271.090
Relative quantity of IL11RA mRNA	23.629	1	0.000 *	0.733	52.176	2.081
IL11RA protein (pg/mL)	26.835	1	0.000 *	0.073	6.966	1.076
Procalcitonin protein (pg/mL)	1.503	1	0.220	−0.032	4.628	0.968
CT finding	64.862	1	0.000 *	139.118	8519.213	2.620 × 10^6^
Ferritin (ng/mL)	5.528	1	0.019 *	0.095	7.335	1.100

* Statistically significant.

## Data Availability

The data presented are available on request by the corresponding authors.

## Supplementary Materials

The following are available online at https://www.mdpi.com/article/10.3390/cells10113098/s1, Figure S1: KEGG ontology pathway database revealed that IL11RA interacts with IL6ST in cytokine signaling and cytokine-cytokine interaction pathway including IL11RA gene interacting with IL6ST, Figure S2: Showing gene ontology of IL11RA mRNA which is involved in cellular response to cytokine stimulus and involved in cytokine mediated signaling pathway that was retrieved from Enrichr tool. Figure S3: Showing the interaction between IL11RA mRNA, hsa-miR-4257 miRNA and lncRNA RP11-773H22.4 lncRNA that was retrieved from enricher tool, Figure S4: Showing gene expression of IL11RA mRNA which is expressed in the whole blood that was retrieved from Gene cards database, Figure S5: Showing gene expression of miR-4257 which is expressed in the whole blood that was retrieved from gene card database, Figure S6: Showing gene expression of lncRNA RP11-773H22.4 which is expressed in the whole blood that was retrieved from gene cards database, Figure S7: Showing that IL11RA mRNA is a direct target of miR-4257 that was retrieved from miRDB database, Figure S8: Showing that IL11RA mRNA is a direct target of miR-4257 that was retrieved from targetscan, Figure S9: Showing sequence alignment between IL11RA mRNA and miR-4257 that was performed using European bioinformatics institute database, Figure S10: Showing sequence alignment between miR-4257 miRNA and RP11-773H22.4 lncRNA that was performed using LncTar tool (**A**), while Figure (**B**) predict the delata free energy for the binding which is −11.82 means that the interaction is thermodynamically favorably between the two sequences, Figure S11: Showing sequence alignment between IL11RA mRNA and RP11-773H22.4 lncRNA that was performed using European bioinformatics institute database, Figure S12: Showing sequence alignment between miR-4257 miRNA and RP11-773H22.4 lncRNA that was performed using European bioinformatics institute database, Figure S13: Showing interaction between lncRNA RP11-773H22.4 and hsa-miR-4257 is thermodynamically favorable that was performed by The Vienna RNA Website. The plot below shows the interaction free energy (RED) Gi and the energy needed to open existing structures in the longer sequence (BLACK) for the target region (top) and the whole range (bottom), Figure S14: Showing interaction between lncRNA RP11-773H22.4 and hsa-miR-4257 is thermodynamically favorable that was performed by The Vienna RNA Website. Results have been computed using RNAup 2.4.18, Table S1: correlation between IL11RA-molecular network, ferritin, procalcitonin and IL-11RA protein.
