# Supplementary material for: In Silico Identification and Clinical Validation of a Novel Long Non-Coding RNA/mRNA/miRNA Molecular Network for Potential Biomarkers for Discriminating SARS CoV-2 Infection Severity
Source: Cells. 2021 Nov 9;10(11):3098. doi: 10.3390/cells10113098 (PMC8625524; doi:10.3390/cells10113098)
Supplement: Supplementary file 1 [file cells-10-03098-s001.zip › cells-1409760-supplementary.pdf]

Supplementary Material

In silico identification and clinical validation of a Novel Long non-coding RNA/mRNA/ miRNA Molecular network as Potential biomarker for discriminating SARS COV-2 infection severity

Sara H.A. Agwa [1,*], Hesham Elghazaly[2], Mahmoud Shawky El Meteini[3] ,Sherif M. Shawky [1,4,5*], Marwa Ali [6], Aya M. Abd Elsamee [7], Safa Matbouly Sayed[8], Nadine Sherif [9] , Howida M. Sharaf [10], Mohamed A.Alhadidy[11] and Marwa Matboli [12]

1 Pathology and Molecular Genomics unit of Medical Ain Shams Research Institute (MASRI), Faculty of Medicine, Ain Shams University, Cairo, 11591, Egypt. sara.h.agwa@med.asu.edu.eg
2 Oncology Department, Medical Ain Shams Research institute (MASRI), Faculty of Medicine, Ain Shams University, Cairo, 11382, Egypt. heshamelghazaly@med.asu.edu.eg
3 Department of General Surgery, the school of Medicine, University of Ain Shams, Cairo, 11591, Egypt; mahmoud_elmeteini@med.asu.edu.eg
4 Biochemistry Department, Faculty of Pharmacy, Misr University for Science and Technology Al-Motamayez District P.O.BOX: 77, 6thOctober City, Giza,12566, Egypt

5 Center of Genomics, Helmy Medical Institute, Zewail City of Science and Technology, Ahmed Zewail Road, October Gardens, 6th of October City, Giza, 12578Egypt; sshawky@zewailcity.edu.eg
6 Medicinal Biochemistry and Molecular biology Department, Faculty of Medicine Ain Shams University, 11591, Cairo, Egypt. drmarwa_matboly@med.asu.edu.eg
7 Biochemistry and Molecular Genomics Unit of Medical Ain Shams Research Institute (MASRI), Ain Shams University, Cairo, 11591, Egypt; aya_ana2025@yahoo.comaya
8 Pediatric Department, School of Medicine, Ain Shams University Hospitals, Cairo 11566, Egypt; safamatboly@yahoo.com
9 Undergraduate student, Faculty of Medicine, Ain Shams University, Abbassia, Cairo, 11591 Egypt; emp17.nadine.s.elzahwy@gmail.com
10 Clinical &molecular pathology, Ain Shams University Hospitals,Cairo, 11591,Egypt howidasharaf@yahoo.com
11 Department of Anesthesiology, Intensive Care & Pain management, Faculty of Medicine, Ain Shams University, Abbassia, Cairo, 11591, Egypt; Mohamed_alaaeldin@med.asu.edu.eg
2 Medicinal Biochemistry and Molecular biology Department, Faculty of medicine, University of Ain Shams, Abbassia, Cairo, Egypt; drmarwa_matboly@med.asu.edu.eg
* Correspondence: sara.h.agwa@med.asu.edu.eg (S.H.A.A.); sshawky@zewailcity.edu.eg (S.M.S.); drmarwa_matboly@med.asu.edu.eg (M.M.)

Supplementary Table S1: Correlation between IL11RA-molecular network, ferritin, procalcitonin and IL11RA protein:

			lncRNA RP11-773H22.4	HSA-MIR-4257	IL11RA mRNA	IL11RA protein (pg/mL)	Procalciton in protein (pg/mL)	Ferritin (ng/ml)
Spearman's Rho	Relative quantity of lncRNA RP11-773H22.4	Correlation Coefficient	1.000	-.475**	.530**	.497**	.210**	.389**
		Sig. (2-tailed)	.	.000	.000	.000	.003	.000
	Relative quantity of HSA-MIR-4257	Correlation Coefficient	-.475**	1.000	-.664**	-.607**	-.327**	-.538**
		Sig. (2-tailed)	.000	.	.000	.000	.000	.000
	Relative quantity of IL11RA mRNA	Correlation Coefficient	.530**	-.664**	1.000	.838**	.449**	.511**
		Sig. (2-tailed)	.000	.000	.	.000	.000	.000
	IL11RA protein(pg/mL)	Correlation Coefficient	.497**	-.607**	.838**	1.000	.482**	.544**
		Sig. (2-tailed)	.000	.000	.000	.	.000	.000
	Procalcitonin protein(pg/mL)	Correlation Coefficient	.210**	-.327**	.449**	.482**	1.000	.264**
		Sig. (2-tailed)	.003	.000	.000	.000	.	.000
	Ferritin(ng/ml)	Correlation Coefficient	.389**	-.538**	.511**	.544**	.264**	1.000
		Sig. (2-tailed)	.000	.000	.000	.000	.000	.

**. Correlation is significant at the 0.01 level (2-tailed)

Supplementary figures

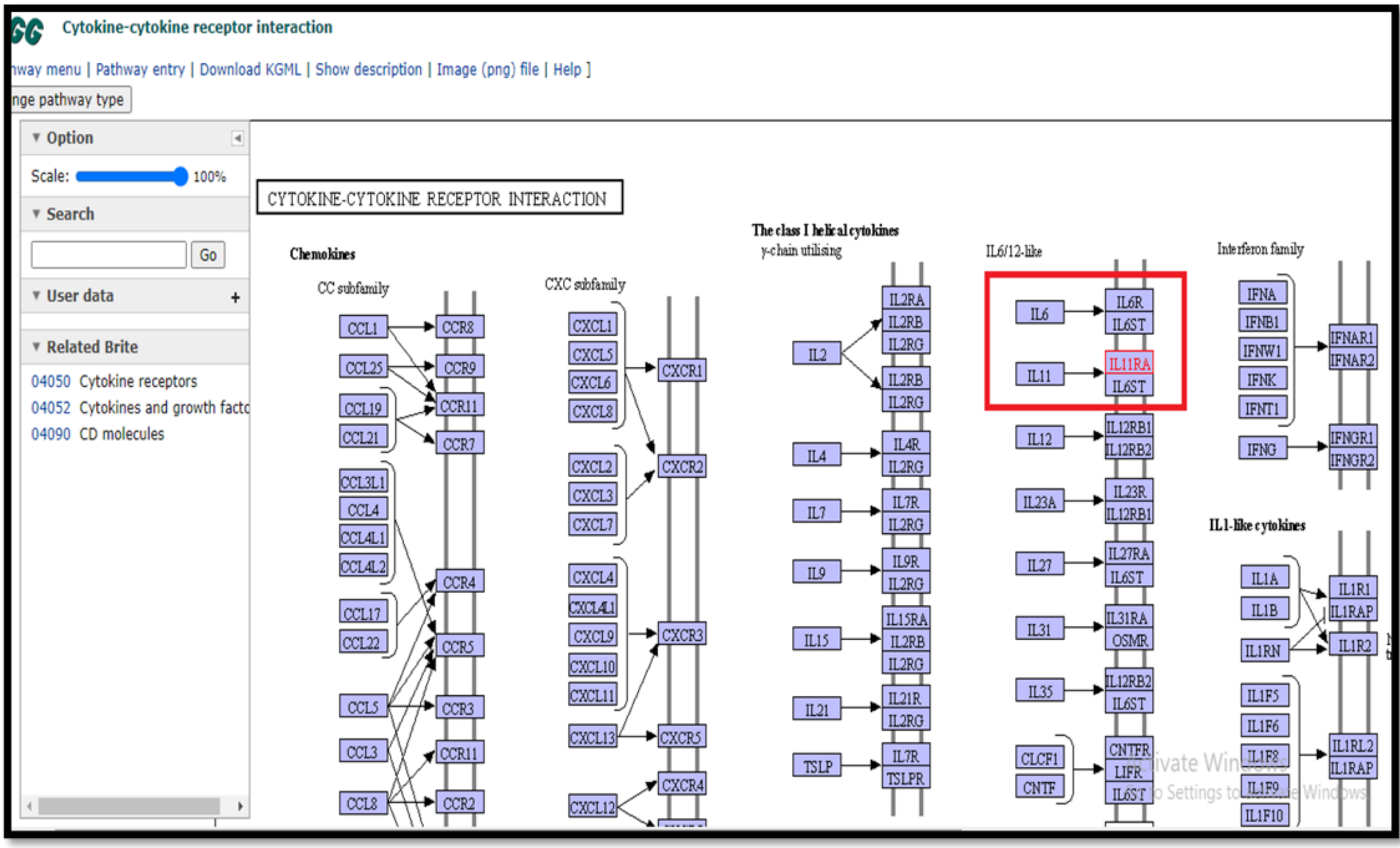

Supplementary figure S1: KEGG ontology pathway database revealed that IL11RA interacts with IL6ST in cytokine signaling and cytokine-cytokine interaction pathway including IL11RA gene interacting with IL6ST. Available at (https://www.genome.jp/kegg/pathway.html)

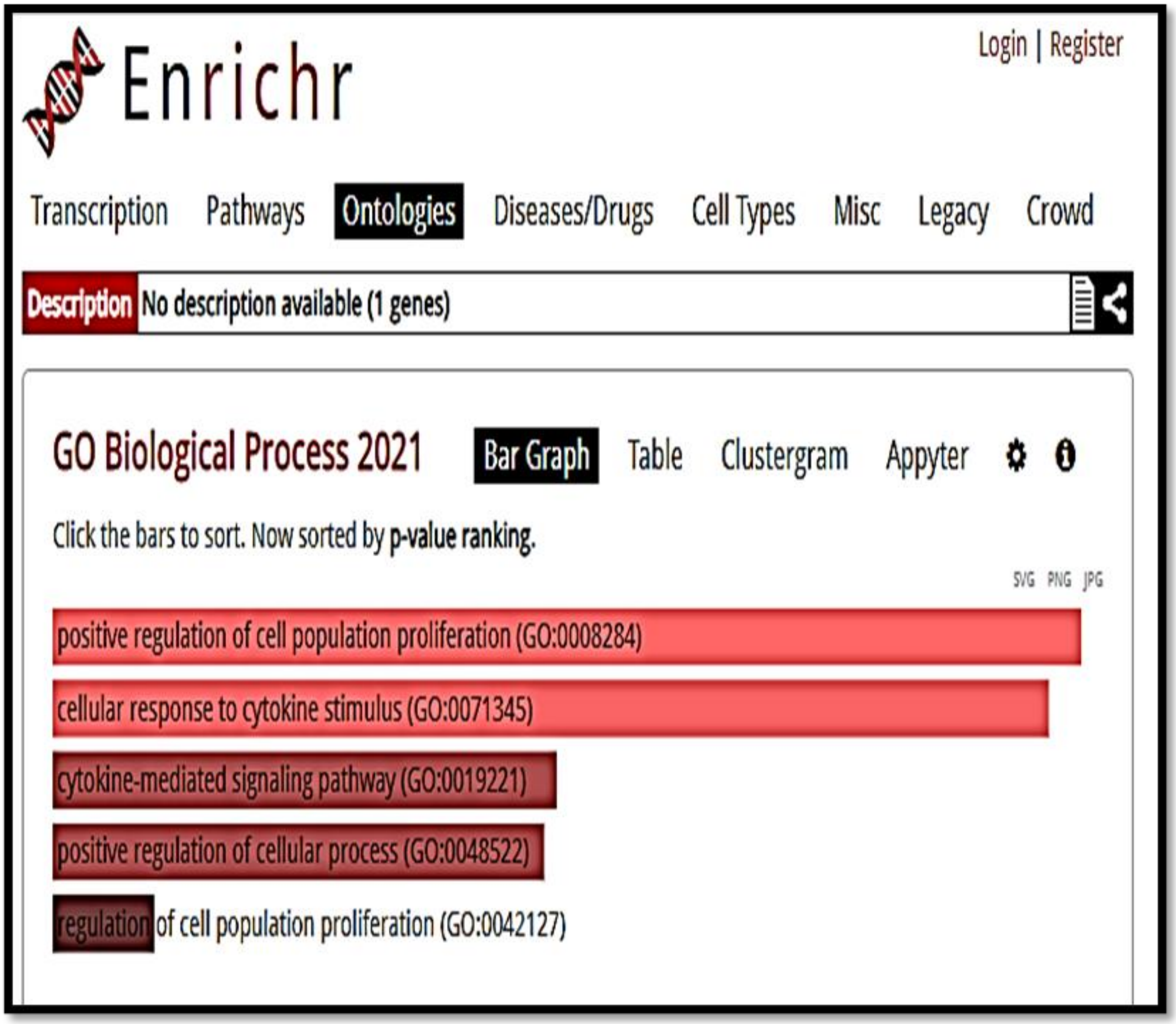

Supplementary figure S2: Showing gene ontology of IL11RA mRNA which is involved in cellular response to cytokine stimulus and involved in cytokine mediated signaling pathway that was retrieved from Enrichr tool.

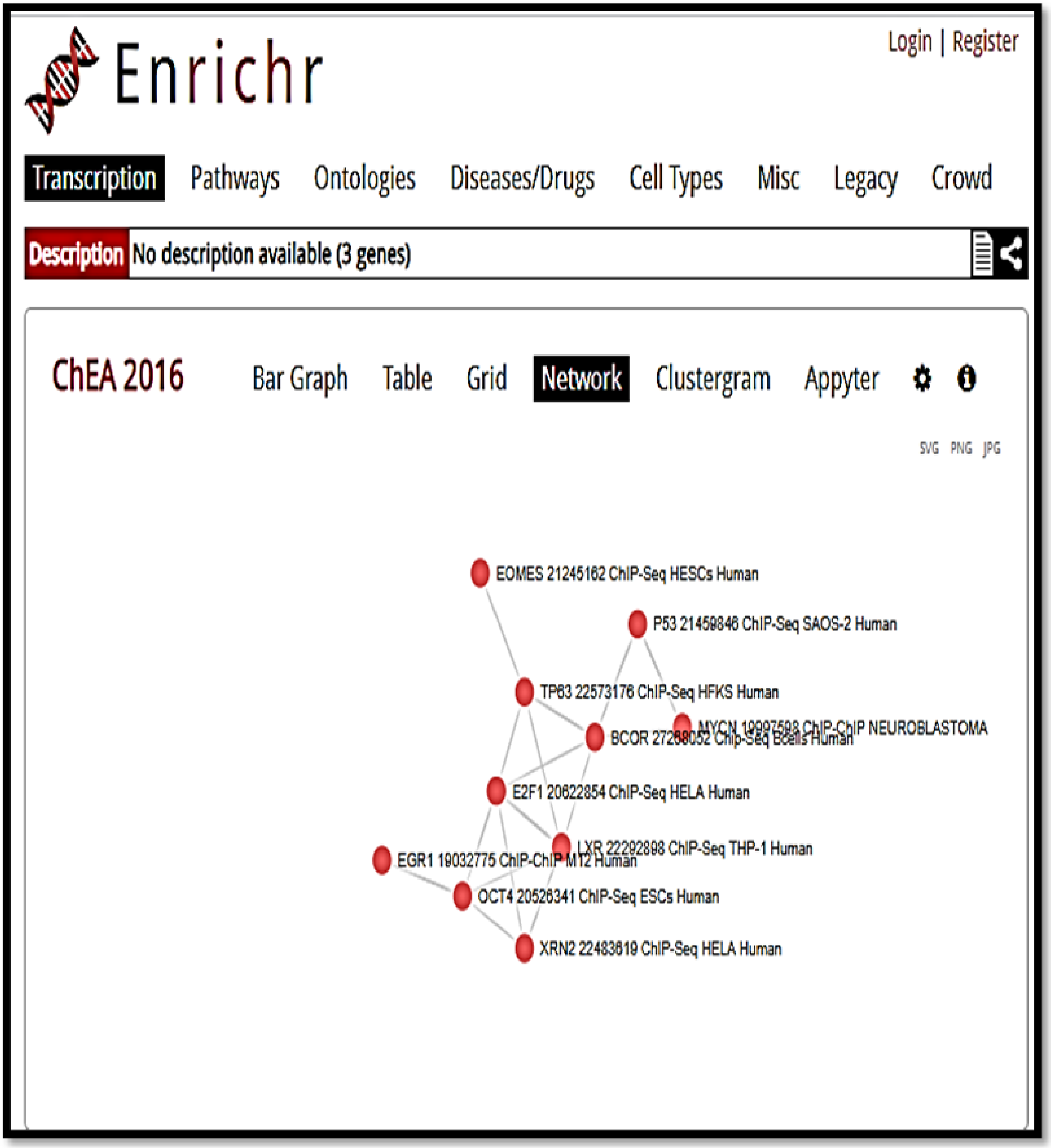

Supplementary figure S3: Showing the interaction between IL11RA mRNA, hsa-miR-4257 miRNA and lncRNA RP11-773H22.4 lncRNA that was retrieved from enricher tool

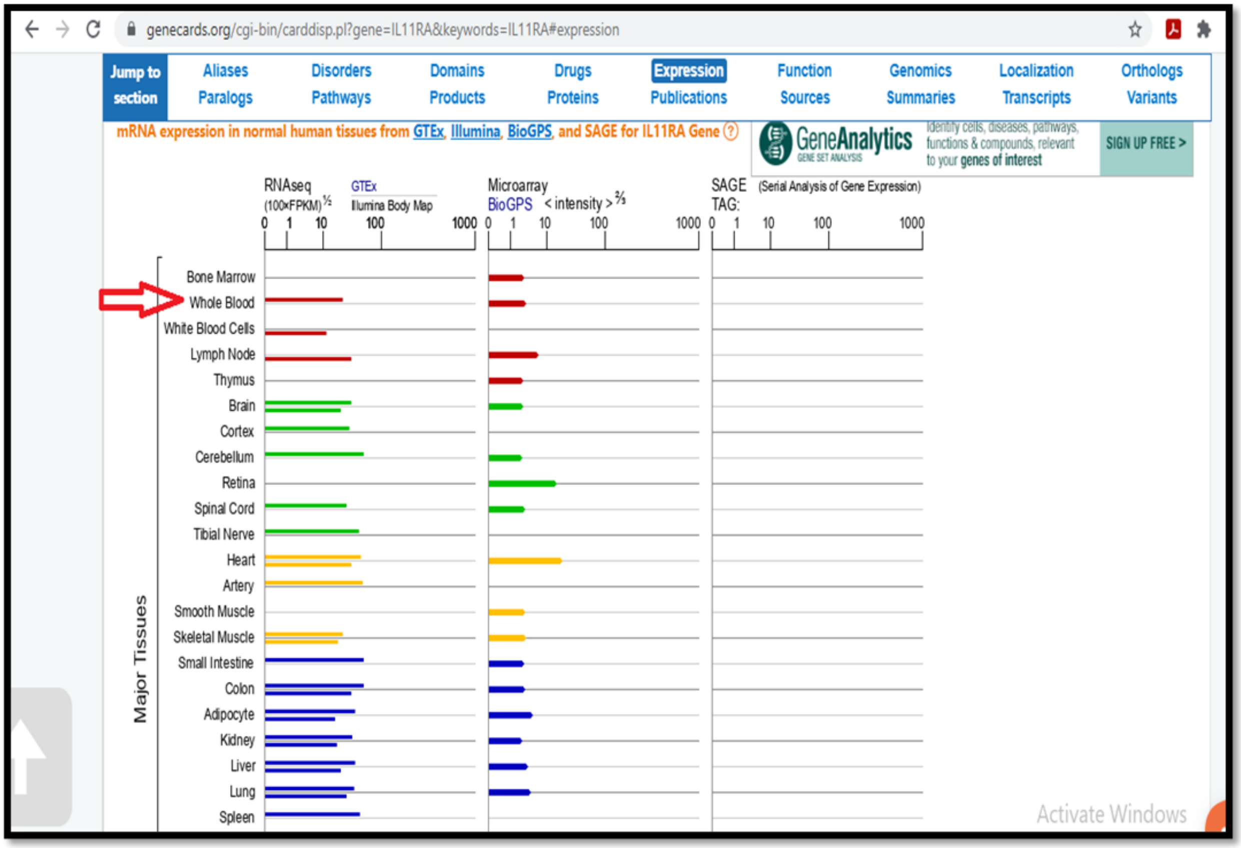

Supplementary figure S4: Showing gene expression of IL11RA mRNA which is expressed in the whole blood that was retrieved from Gene cards database Available at (https://www.genecards.org/cgi-bin/carddisp.pl?gene=IL11RA&keywords=IL11RA#expression

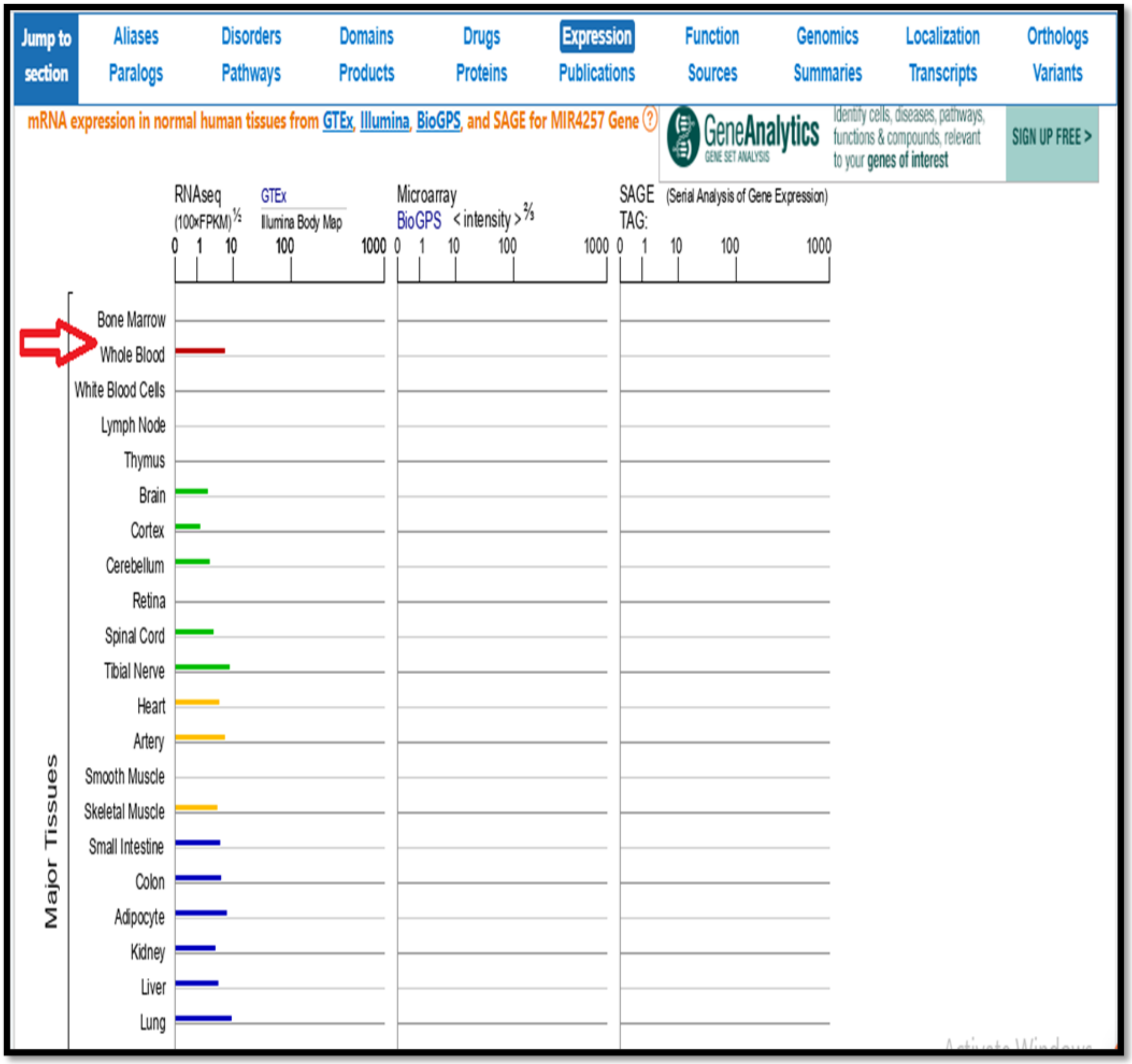

Supplementary figure S5: Showing gene expression of miR- 4257 which is expressed in the whole blood that was retrieved from gene card database (https://www.genecards.org/cgi-bin/carddisp.pl?gene=MIR4257&keywords=hsa%5C-miR%5C-4257)

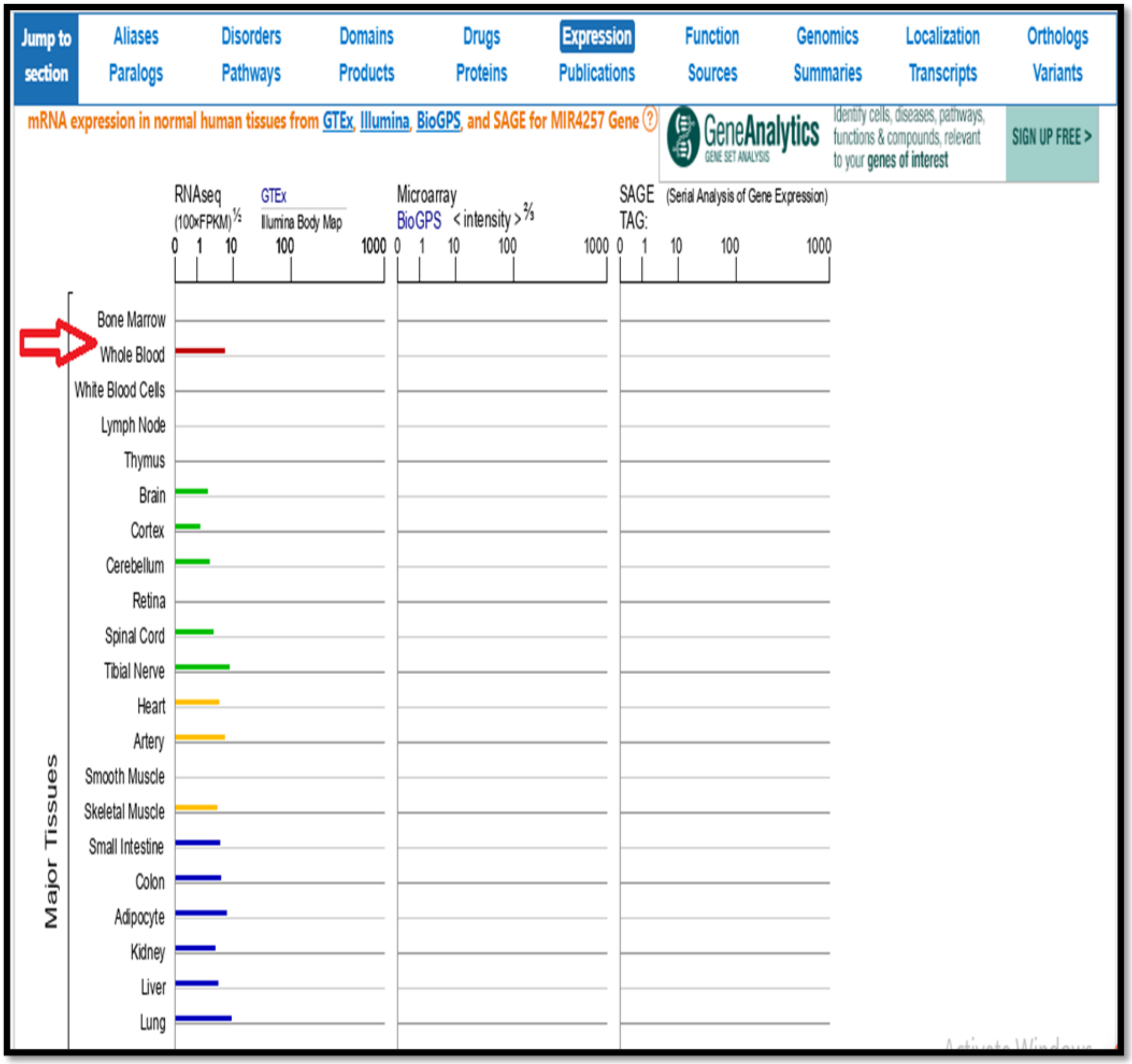

Supplementary figure S6: Showing gene expression of lncRNA RP11-773H22.4 which is expressed in the whole blood that was retrieved from gene cards database available at (https://www.genecards.org/cgi-bin/carddisp.pl?gene=ENSG00000266969&keywords=lnc%5C-PTPN2%5C-6#expression

Gene '3590' is predicted to be targeted by 19 miRNAs in miRDB.

Target Detail	Target Rank	Target Score	miRNA Name	Gene Symbol	Gene Description
Details	1	91	hsa-miR-4753-3p	IL11RA	interleukin 11 receptor subunit alpha
Details	2	77	hsa-miR-6810-3p	IL11RA	interleukin 11 receptor subunit alpha
Details	3	77	hsa-miR-6801-3p	IL11RA	interleukin 11 receptor subunit alpha
Details	4	75	hsa-miR-4696	IL11RA	interleukin 11 receptor subunit alpha
Details	5	73	hsa-miR-4257	IL11RA	interleukin 11 receptor subunit alpha
Details	6	67	hsa-miR-1226-3p	IL11RA	interleukin 11 receptor subunit alpha
Details	7	66	hsa-miR-4643	IL11RA	interleukin 11 receptor subunit alpha
Details	8	62	hsa-miR-6809-3p	IL11RA	interleukin 11 receptor subunit alpha
Details	9	62	hsa-miR-603	IL11RA	interleukin 11 receptor subunit alpha
Details	10	60	hsa-miR-3941	IL11RA	interleukin 11 receptor subunit alpha
Details	11	60	hsa-miR-12123	IL11RA	interleukin 11 receptor subunit alpha
Details	12	59	hsa-miR-8485	IL11RA	interleukin 11 receptor subunit alpha
Details	13	59	hsa-miR-4717-3p	IL11RA	interleukin 11 receptor subunit alpha
Details	14	58	hsa-miR-7155-5p	IL11RA	interleukin 11 receptor subunit alpha
Details	15	56	hsa-miR-519d-5p	IL11RA	interleukin 11 receptor subunit alpha
Details	16	56	hsa-miR-3678-3p	IL11RA	interleukin 11 receptor subunit alpha
Details	17	56	hsa-miR-12114	IL11RA	interleukin 11 receptor subunit alpha
Details	18	54	hsa-miR-6747-3p	IL11RA	interleukin 11 receptor subunit alpha
Details	19	53	hsa-miR-5695	IL11RA	interleukin 11 receptor subunit alpha

Supplementary figure S7: Showing that IL11RA mRNA is a direct target of miR- 4257 that was retrieved from miRDB database (http://mirdb.org/

	Predicted consequential pairing of target region (top) and miRNA (bottom)	Site type	Context++ score	Context++ score percentile	Weighted context++ score	Conserved branch length	P_{CT}
Position 170-177 of IL11RA 3' UTR<br>hsa-miR-4257	5' ...CUGAAAGGUGCUUGUACCUCUGA...<br>||||||| 3' GAGUCAGGGGUGGAGACC	8mer	-0.44	99	-0.44	0	N/A

Supplementary figure S8: Showing that IL11RA mRNA is a direct target of miR- 4257 that was retrieved from targetscan database available at (http://www.targetscan.org/cgi-bin/targetscan/vert_72/view_gene.cgi?rs=ENST00000318041.9&taxid=9606&members=miR-4257&showcnc=0&shownc=0&showncf1=1&showncf2=1&subset=1

```
Input form | Web services | Help & Documentation | Bioinformatics Tools FAQ

EMBOSS_001        1 --------------------------------------------------      0

EMBOSS_001      301 AGACAGCACTGATGAGGGCACCTACATCTGCCAGACCCTGGATGGTGCAC    350

EMBOSS_001        1 --------------------------------------------------      0

EMBOSS_001      351 TTGGGGGCACAGTGACCCTGCAGCTGGGCTACCCTCCAGCCCGCCCTGTT    400

EMBOSS_001        1 --------------------------------------------------      0

EMBOSS_001      401 GTCTCCTGCCAAGCAGCCGACTATGAGAACTTCTCTTGCACTTGGAGTCC    450

EMBOSS_001        1 --------------------------------------------------      0

EMBOSS_001      451 CAGCCAGATCAGCGGTTTACCCACCCGCTACCTCACCTCCTACAGG--AA    498
                                                                ||  .|
EMBOSS_001        1 --------------------------------------------GGCUUA      6

EMBOSS_001      499 GAAGACAGT-CCTAGGAGCTGATAGCCAGA-----GGAGGAGTCCATCCA    542
                    ||| ||||: ||:|||      :||   ||     ||||||| |.|   |
EMBOSS_001        7 GAA-ACAGUCCCUAGG------UAG---GAUUUGGGGAGGAG-CUA---A     42

EMBOSS_001      543 CAGGGCCCT---GGCCATGCCCACAGGATCCCCTAGGGGCTGCCCGCTGT    589
                    .|.|.|||:   ||    |||||.|||      :.|||.|:|..| |:..
EMBOSS_001       43 GAAGCCCCUACAGG----GCCCAGAGG------UGGGGACUGAGC-CUUA     81

EMBOSS_001      590 GTTGTCCACGGGGCTGAGTTCTGGAGCCAGTACCGGATTAATGTGACTGA    639
                    |::|.
EMBOSS_001       82 GUUGG---------------------------------------------     86

EMBOSS_001      640 GGTGAACCCACTGGGTGCCAGCACACGCCTGCTGGATGTGAGCTTGCAGA    689

EMBOSS_001       87 --------------------------------------------------     86

EMBOSS_001      690 GCATCTTGCGCCCTGACCCACCCCAGGGCCTGCGGGTAGAGTCAGTACCA    739

EMBOSS_001       87 --------------------------------------------------     86

EMBOSS_001      740 GGTTACCCCCGACGCCTGCGAGCCAGCTGGACATACCCTGCCTCCTGGCC    789
```

Supplementary figure S9: Showing sequence alignment between IL11RA mRNA and miR-4257 that was performed using European bioinformatics institute database available at (https://www.ebi.ac.uk/Tools/services/web/toolresult.ebi?jobId=emboss_needle-I20210805-231255-0164-87398889-p1m)

(A)

```
Query	Length_Query	Target	Length_Target	dG	ndG	Start_Position_Query	End_Position_Query
Start_Position_Target	End_Position_Target
RP11-773H22.4	474	hsa-mir-4257	86	-11.82	-0.1515	3	88	1	86
5'
TTTGAAGCGAGGAAGCCGCGCCCTGCGAGTCCGGAGGAGGAAATGGAGCTCTGCGCTGCGTGGGACCCGGGGACGGCTCCCGTTGGCGCCGCCTTCAAGGTAAGGGATTAAGAGGCCGAGAGAGATATCGTCTGCGAGGGTGAGGATACTGGAGGTTGGCAGTGTCAGCATCGCAAGAGTGCTCT
ACCAGAGGAGGAGGAGGAGGAAGGAGCTGGGCATAACTGGACCATCTCGATCCAGCCCGTGGGGAATCCAGAGGGGATAACAAGAGGATATAACCACAGGACAATTCATTAGGCTGTGTTTTGACGTTTCCTGAATTCTATTCCAAATTATTCACAAAGGCATATTAAAATAATTTTATCTTACA
AATTCACATATTTAAAATTTGAACATGGAAACAGACTCTAGATGACATCAAATATTCCTTATTATGATATACAAGAATGTAACATATGCTAAATATTC 3'
    ..||...|||..........|||......||......||......||||............|||..|||......||......
 3' GGTTGATTCCGAGTCAGGGGTGGAGACCCGGGACATCCCCGAAGAATCGAGGAGGGGTTTAGGATGGATCCCTGACAAAGATTCGG 5'
```

(B)

Query	Length_Query	Target	Length_Target	dG	ndG	Start_Position_Query	End_Position_Query	Start_Position_Target	End_Position_Target
RP11-773H22.4	474	hsa-mir-4257	86	-11.82	-0.1515	3	88	1	86

Supplementary figure S10: Showing sequence alignment between miR- 4257 miRNA and RP11-773H22.4 lncRNA that was performed using LncTar tool (A), while Figure (B) predict the delata free energy for the binding which is -11.82 means that the interaction is thermodynamically favorably between the two sequences. (http://www.cuilab.cn/files/lnctar/pdm_result_202185172654.txt)

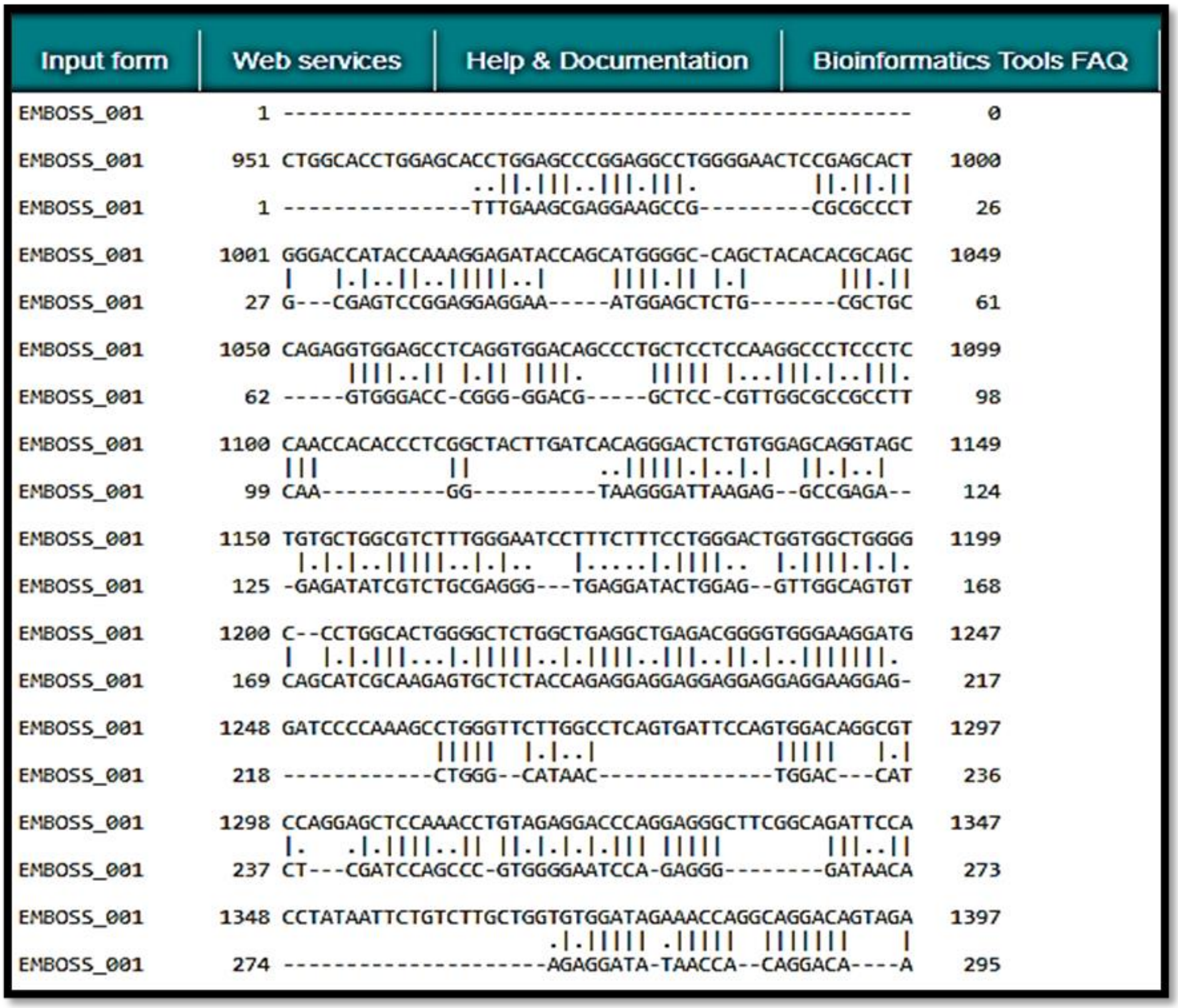

Supplementary figure S11: Showing sequence alignment between IL11RA mRNA and RP11-773H22.4 lncRNA that was performed using European bioinformatics institute database available at (https://www.ebi.ac.uk/Tools/services/web/toolresult.ebi?jobId=emboss_needle-I20210805-231838-0992-90221444-p2m)

```
EMBOSS_001         1 TTTGAAGCGAGGAAGCCGCGCGCCCT--GCGAGTCCGGAGGAGGA--AAT     46
                          .||...|||.|.|   .|||.  |...|....|.||||||  .|.
EMBOSS_001         1 -----GGCUUAGAAACAG---UCCCUAGGUAGGAUUUGGGGAGGAGCUAA     42

EMBOSS_001        47 GGAGCTCTGCGCTGCGTGGACCCG----GGGGACGGCTCC--CGTTGGC     90
                     |.|||.|    |..| .|||.||.|    ||||||.|..||   .|..||
EMBOSS_001        43 GAAGCCC----CUAC-AGGGCCCAGAGGUGGGGACUGAGCCUUAGUUGG-     86

EMBOSS_001        91 GCCGCCTTCAAGGTAAGGGATTAAGAGGCCGAGAGAGATATCGTCTGCGA    140

EMBOSS_001        87 --------------------------------------------------     86

EMBOSS_001       141 GGGTGAGGATACTGGAGGTTGGCAGTGTCAGCATCGCAAGAGTGCTCTAC    190
```

Supplementary figure S12: Showing sequence alignment between miR- 4257 miRNA and RP11-773H22.4 lncRNA that was performed using European bioinformatics institute database available at (https://www.ebi.ac.uk/Tools/services/web/toolresult.ebi?jobId=emboss_needle-I20210805-232348-0040-53372138-p2m)

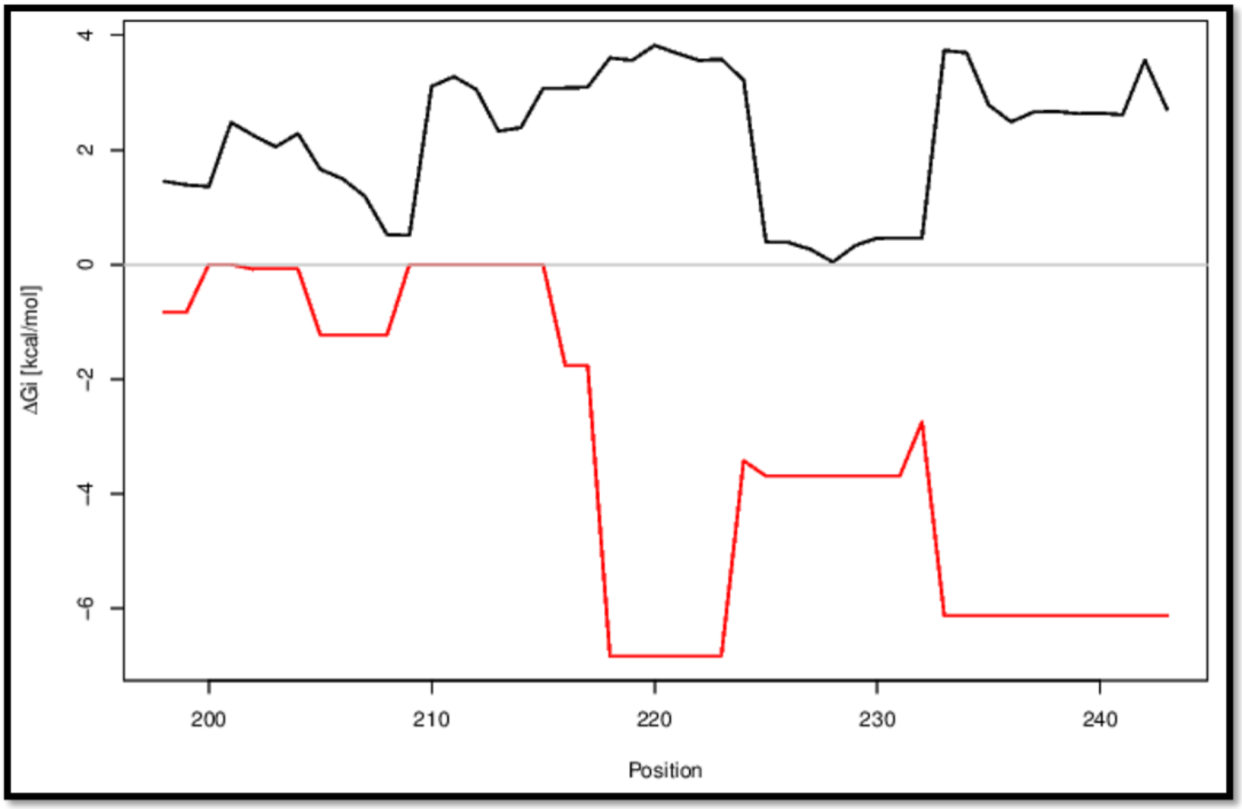

Supplementary figure S13: Showing interaction between lncRNA RP11-773H22.4 and hsa-miR-4257 is thermodynamically favorable that was performed by The Vienna RNA Website. The plot below shows the interaction free energy (RED) Gi and the energy needed to open existing structures in the longer sequence (BLACK) for the target region (top) and the whole range (bottom).

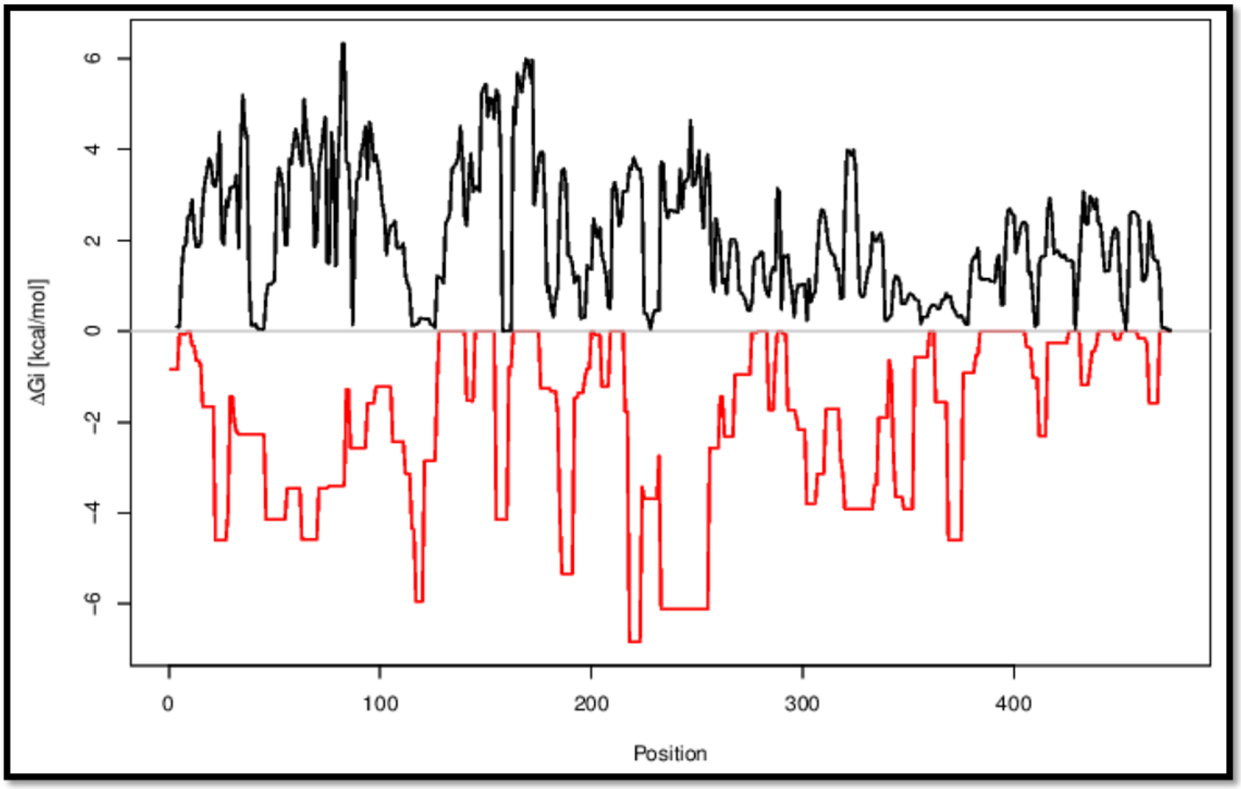

Supplementary figure S14: Showing interaction between lncRNA RP11-773H22.4 and hsa-miR-4257 is thermodynamically favorable that was performed by The Vienna RNA Website. Results have been computed using RNAup 2.4.18.
